# OPTIMATRIX v2.0: Optimised protocol to mitigate microbial blooms in the micro-Matrix bioreactor platform used as an *ex vivo* human distal colon model

**DOI:** 10.1016/j.mex.2025.103275

**Published:** 2025-03-22

**Authors:** Arghya Mukherjee, Nicola Ferremi Leali, Elisa Salvetti, Sandra Torriani, Paul D. Cotter, Harsh Mathur

**Affiliations:** a Teagasc Food Research Centre, Moorepark, Fermoy, Cork, Ireland; b APC Microbiome Ireland, Cork, Ireland; c VistaMilk, Fermoy, Co., Cork, Ireland; d Department of Biotechnology, University of Verona, Italy; e VUCC-DBT (Verona University Culture Collection, Dept. of Biotechnology), University of Verona, Italy

**Keywords:** Gut microbiome, Distal colon model, Faecal microbiota, *Ex vivo* model, Micro-Matrix, Microbial blooms, 16S rRNA amplicon sequencing, Flow cytometry, Relative abundance, Absolute abundance, OPTIMATRIX v2.0. Optimised methods to minimise microbial blooms, maintain microbial diversity and improve reproducibility in the micro-Matrix bioreactor platform used as an *ex vivo* batch model of the human distal colon

## Abstract

We previously reported optimisation of the methodology to mitigate *Escherichia coli* blooms and associated loss of microbial diversity when using the micro-Matrix bioreactor platform as an *ex vivo* model of the human distal colon. Here, we provide further critical insights that we have gained in this regard through follow-up experiments. We tested four separate faecal fermentation media compositions for the purposes of such *ex vivo* distal colon model experiments and found that the media composition described by MacFarlane *et al.* is the most suitable for mitigating such microbial blooms, and concurrently, maintaining microbial diversity. We also tested if pooled or individual donor faecal samples were more suitable and found that pooled samples performed better in terms of maintaining gut microbiota diversity in such batch culture model experiments using the micro-Matrix system. Finally, we determined that prolonged experiments, i.e. for durations of up to 96 h, may be warranted with a view to affording particularly fastidious gut microbes an opportunity to grow and compete with their less fastidious counterparts. Essentially, we provide critical insights into:

• Optimal faecal fermentation media to minimise blooms and preserve diversity in *ex vivo* colon model experiments
• Optimal faecal inoculum source and duration of experiments.

## Graphical abstract

**Figure:**
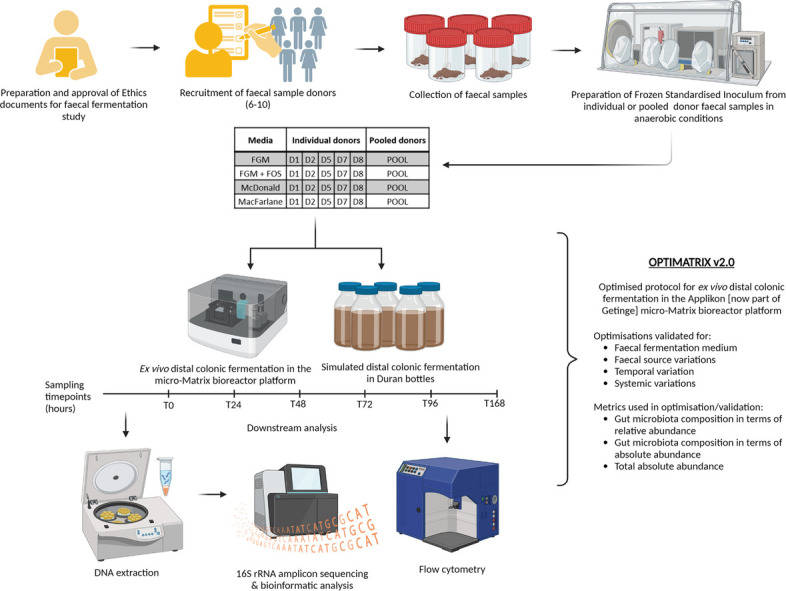

Specifications table

**Table:** 

Subject area:	Immunology and Microbiology
More specific subject area:	Human gut microbiome
Name of your method:	OPTIMATRIX v2.0. Optimised methods to minimise microbial blooms, maintain microbial diversity and improve reproducibility in the micro-Matrix bioreactor platform used as an *ex vivo* batch model of the human distal colon.
Name and reference of original method:	Further development in methods to attenuate microbial blooms associated with *Escherichia* and other genera as described in Mathur et al. 2023 [1] with a specific focus on testing different faecal fermentation media formulations, duration of colon model experiments, and faecal slurry inocula derived from either pooled or individual donor samples.
Resource availability:	Equipment includes the micro-Matrix bioreactor fermentation system (Getinge; formerly Applikon). The data for this study have been deposited in NCBI with BioProject ID PRJNA1191888. (http://www.ncbi.nlm.nih.gov/bioproject/1191888)

## Background

Several reliable continuous and semi-continuous human distal colon models have been reported over the last two decades [2,3]. While perhaps being somewhat laborious to operate with large fermentation volumes and associated lengthy fermentation times, the general consensus is that continuous and semi-continuous models are more reliable than batch models, as they take into account the replenishment of the faecal fermentation media with concurrent efflux of waste by-products. The batch culture-based micro-Matrix bioreactor platform however, provides an advantage over these continuous/semi-continuous human distal colon models in being a low-volume, high-throughput system.

Although highly useful in terms of its high-throughput ability to simulate the human gut microbiota using faecal inocula, the batch culture nature of the system makes it prone to *Escherichia coli, Enterococcus faecalis* and *Klebsiella* spp. blooms (i.e., drastic increase in relative abundance). Such microbial blooms are often coupled with a concomitant loss of microbial diversity. We previously reported the phenomenon of a drastic increase in *E. coli* when using the micro-Matrix bioreactor platform in conjunction with a frozen standardised inoculum (FSI) consisting of a pooled faecal slurry and faecal fermentation media developed by Fooks and Gibson [1,4]. Since such microbial blooms and the concurrent reduction in microbial diversity is unlikely to be representative of the *in vivo* gut microbiota in the human distal colon, it has remained a significant obstacle in accurately evaluating the impact of various test substrates, including diverse foodstuffs, on the gut microbiome using the micro-Matrix platform.

In the present study, we have assessed the impact of several factors that are likely to contribute to such microbial blooms and attempted to find solutions to mitigate the same, whilst concurrently maintaining microbial diversity, which is a characteristic of a healthy gut microbiota under normal physiological conditions. The primary hypotheses we attempted to test in this study involved evaluating the following factors:

(i). *Inoculum source*: is pooled FSI more/less prone to microbial blooms and loss of diversity than FSI generated from individual donor faecal samples?
(ii). *Faecal fermentation medium:* does the choice of faecal fermentation medium have an impact on microbial blooms and associated loss of diversity?
(iii). *Duration of the colon model experiment:* whether or not such *ex vivo* distal colon model experiments conducted for longer durations might mitigate microbial blooms, thereby restoring diversity.
(iv). *Systemic factors:* are such blooms solely associated with the micro-Matrix system or do such trends occur in similar batch culture experiments conducted in Duran bottles?

To this end, we conducted *ex vivo* distal colon model experiments for a duration of 168 hours in both static Duran glass bottles in anerobic chambers as well as the micro-Matrix bioreactor platform using faecal inocula sourced from different individuals or a pooled faecal inoculum. Samples retrieved from the experimental setups at intervals of 24 hours were subjected to 16S rRNA-based amplicon sequencing and flow cytometry to determine the relative and absolute abundances of microbial taxa respectively, which in turn were used to validate OPTIMATRIX v2.0, i.e. the optimised method.

## Method details

### OPTIMATRIX v2.0: optimised micro-Matrix SOP for use as an *ex vivo* human distal colon model

Here we outline the optimisation steps for conducting *ex vivo* distal colon model experiments using the micro-Matrix bioreactor system with important recommendations, including the use of an optimised Faecal Fermentation Media derived from MacFarlane et al. [5] (referred to as MCF hereafter), the use of pooled Frozen Standardised Inoculum (FSI) and conducting the micro-Matrix experiment for a longer duration of at least 96 h. In the checklist below, we provide recommendations for volumes of test substrates to be added, examples of which may include liquid food or reconstituted food compounds. We do however advise users to further optimise such volumes based on their own experimental design. All optimised media compositions and associated calculations for micro-Matrix experiments, whereby we take into account the ratio of the test substrate, the MCF and the FSI are provided in Table 1.

1). The optimised media formulation for *ex vivo* human distal colon model experiments using the micro-Matrix system is a modified version of the media previously reported by MacFarlane et al. [5,6] and is outlined in Table 1.
2). Make 1 Litre of this media by following the calculations described in Table 1. Autoclave at 121°C X 15 min. After autoclaving, cool the media to room temperature. Prepare the required quantity of cysteine-HCl by weighing out the powder and reconstituting in 5 ml of sterile water. Filter-sterilise the solution using a 0.22 µm filter and add it aseptically to the autoclaved Duran bottle of media after the media has cooled to room temperature. Aliquot the final 1 L into 170 ml volumes in 250 ml Duran bottles. The preparation of aliquots aseptically is critical to minimise the chances of contamination upon opening and closing of the lids. Furthermore, 250 ml Duran bottles would be suitable for this purpose to minimise the head space in the bottles, thereby facilitating the maintenance of strict anaerobic conditions inside the anaerobic hood. After aliquoting, store the Duran bottles at 4 °C for a maximum of one week to minimise chances of contamination. This is particularly important due to the rich composition of the media.
3). Conduct the micro-Matrix checklists as outlined in Mathur et al. [1] with some further optimisations described below.
4). Thaw a 12 ml aliquot of Frozen Standardised Inoculum (FSI) derived from pooled faecal samples for 10 min inside an anaerobic hood at 37 °C.
5). Add this 12 ml aliquot of FSI to 170 ml of the modified MacFarlane media (MCF) and mix by gently inverting 6 times.
6). Dispense 7 ml of the MCF media-FSI mix to each of the 24 wells in a micro-Matrix cassette.
7). Add 1 ml of the test substrate to the 7 ml mix, resulting in 8 ml volumes in each of the 24 wells. For the Faecal Fermentation Media negative controls, add 1 ml of additional MCF media to keep the volumes consistent in all wells. This starting point is referred to as T0 (zero hours).
8). Take 2 × 1 ml aliquots from each well for this T0 time point prior to commencing the fermentation. This will result in a final volume of 6 ml in each well at the start of fermentation. Approximately 16 hours before the next time point, place a fresh bottle of the MCF media inside the anaerobic hood to pre-equilibrate to anaerobic conditions. Conduct this step on a daily basis prior to the relevant time points.
9). After 24 h of fermentation, take 3 × 1 ml aliquots from each well. This will reduce the total volume in each well to 3 ml. Replenish each well with 2 ml of fresh MCF media and 1 ml of the test substrate. This will restore the total volume to 6 ml in each well. Taking 3 × 1 ml aliquots will partially remove any toxic metabolites which may have built up during the fermentation, while replenishing with fresh media and substrates mimics *in vivo* physiological conditions and affords the faecal microbiota an opportunity to utilise these substrates to grow. For the FFM negative controls, replenish the relevant wells with 3 ml of fresh MCF media.
10). Repeat the above step for 48 h (T48), 72 h (T72), 96 h (T96) and any other time points in the experiment.

**Table 1:** Detailed composition for various faecal fermentation media (FFM) used in the study.

Reagent	Modified MacFarlane medium [5]		Modified McDonalds medium [7]		Fooks and Gibson medium [4]		Modified Fooks and Gibson medium [4]	
	Per Litre (g/L or ml/L)	With test substrates [7 ml media + 1 ml test substrate + FSI] (g/L)	Per Litre (g/L or ml/L)	With test substrates [7 ml media + 1 ml test substrate + FSI] (g/L)	Per Litre (g/L or ml/L)	With test substrates [7 ml media + 1 ml test substrate + FSI] (g/L)	Per Litre (g/L or ml/L)	With test substrates [7 ml media + 1 ml test substrate + FSI] (g/L)
Arabinogalactan	-	-	2	2.457235	-	-	-	-
Bile salts	0.4	0.491447	0.5	0.61430875	0.5	0.614255	0.5	0.614255
CaCl_2_	0.15	0.184292625	0.01	0.012286175	-	-	-	-
Casein	3	3.6858525	3	3.6858525	-	-	-	-
FeSO_4_.7H_2_O	0.005	0.006143088	-	-	-	-	-	-
Fructooligosaccharide	-	-	-	-	-	-	25	30.71275
Guar Gum	1	1.2286175	-	-	-	-	-	-
Hemin	0.05	0.061430875	0.005	0.006143088	0.05 g (dissolved in 3 drops of 1 M NaOH)	0.061426	0.05 g (dissolved in 3 drops of 1 M NaOH)	0.061426
Inulin	1	1.2286175	1	1.2286175	-	-	-	-
K_2_HPO_4_	0.5	0.61430875	0.04	0.0491447	-	-	-	-
KH_2_PO_4_	-	-	0.04	0.0491447	-	-	-	-
KCl	4.5	5.52877875	-	-	-	-	-	-
L-cysteine HCl	0.8	0.982894	0.5	0.61430875	1	1.22851	1	1.22851
Menadione (Vitamin K3)	-	-	0.001	0.001228618	-	-	-	-
MgSO_4_	1.25	1.535771875	0.01	0.012286175	-	-	-	-
Mucin	4	4.91447	4	4.91447	-	-	-	-
NaCl	4.5	5.52877875	0.1	0.12286175	-	-	-	-
NaHCO_3_	1.5	1.84292625	2	2.457235	2	2.45702	2	2.45702
Pectin	2	2.457235	2	2.457235	-	-	-	-
Peptone water	5	6.1430875	2	2.457235	-	-	-	-
Phytonadione (Vitamin K1)	-	-	-	-	10µl	0.012285	10µl	0.012285
Starch	5	6.1430875	5	6.1430875	-	-	-	-
Tryptone	5	6.1430875	-	-	-	-	-	-
Tryptone water	-	-	-	-	2	2.45702	2	2.45702
Tween 80	1	1.2286175	-	-	0.002	0.002457	0.002	0.002457
Xylo-oligosaccharide	-	-	2	2.457235	-	-	-	-
Yeast extract	4.5	5.52877875	2	2.457235	2	2.45702	2	2.45702
NaCl (10% w/v autoclaved stock solution)	-	-	-	-	1ml/L	1.22851	1ml/L	1.22851
KH_2_PO_4_ (4% w/v autoclaved stock solution)	-	-	-	-	1ml/L	1.22851	1ml/L	1.22851
K_2_HPO_4_ (4%w/v autoclaved stock solution)	-	-	-	-	1ml/L	1.22851	1ml/L	1.22851
CaCl_2_.6H_2_0 (4% w/v autoclaved stock solution)	-	-	-	-	1ml/L	1.22851	1ml/L	1.22851
MgSO_4_.7H_2_0 (1% w/v autoclaved stock solution)	-	-	-	-	1ml/L	1.22851	1ml/L	1.22851

## Method validation

**Fig. 1:**
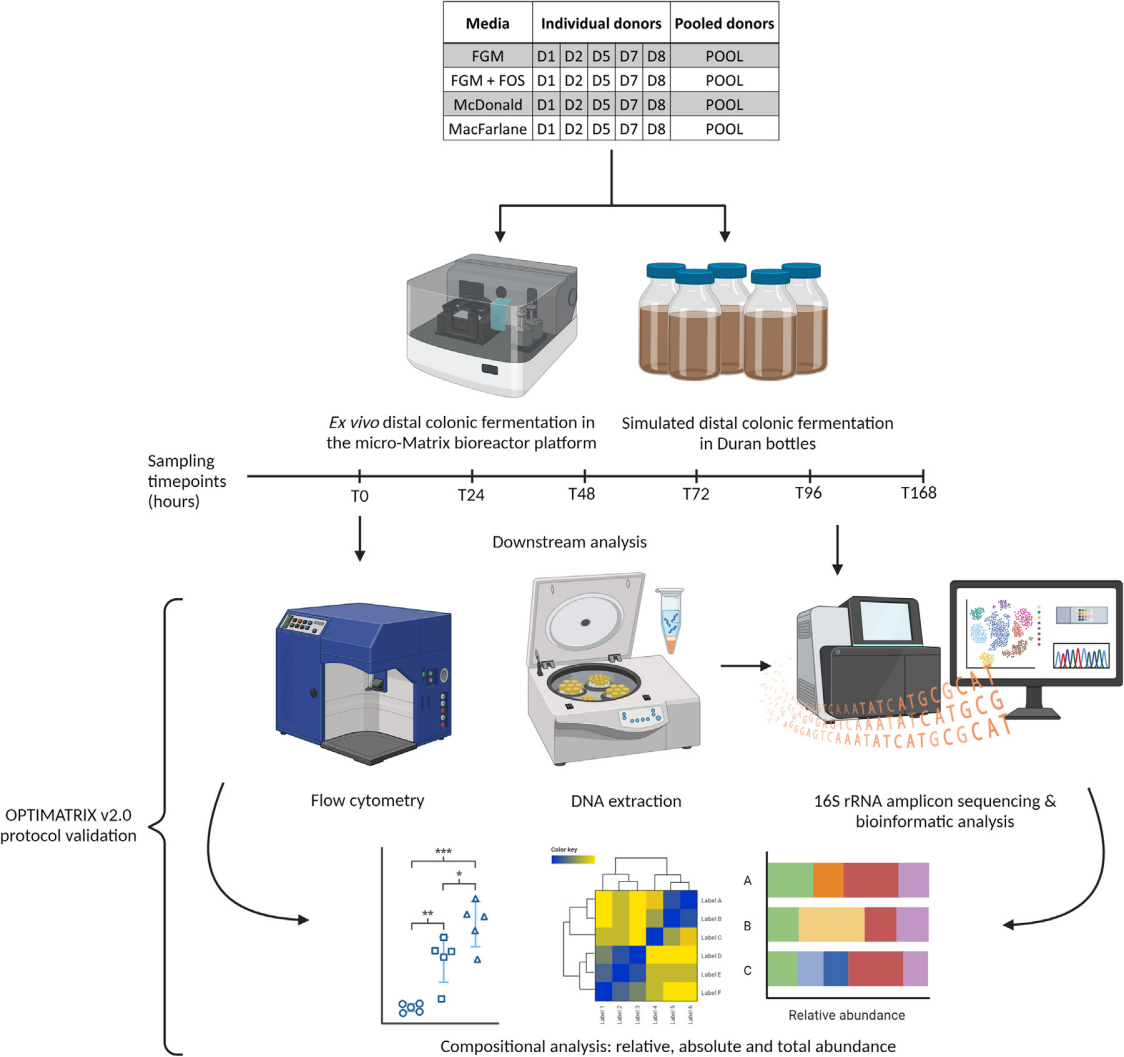
OPTIMATRIX v2.0 pipeline and validation schema. The figure presents a simplified schematic of the pipeline followed to test the various hypotheses that fed into OPTIMATRIX v2.0 standardisation and validation. Subsequent to ethics approval, faecal samples were collected from individual donors (D1-D8) from which pooled donor frozen standardised inoculum (FSI) was made along with individual donor FSIs. The pooled and individual donor FSIs were then used as inocula for a week-long experiment simulating the distal colon in both Duran bottles and the micro-Matrix bioreactor platform. To this end, the inocula were used in conjunction with different media (FGM, Fooks & Gibson media [1,4]; FGMFOS, Fooks & Gibson media supplemented with 2.5% w/v FOS; MCF, Modified media derived from MacFarlane et al. [5]; MCD, Modified media derived from McDonald et al. [7]) and the faecal fermentations sampled with corresponding media replacement every 24 hours. Aliquoted samples were then subjected to DNA extraction and 16S rRNA-based amplicon sequencing to determine microbial taxonomic compositional relative abundance. Aliquoted samples were also processed using flow cytometry to obtain absolute cellular abundances, which were used as secondary validation endpoints. Heatmaps, scatter plots etc shown here are representative of the graphs generated in the study and are not directly corroborative. Created in https://BioRender.com.

In the present study, we validated OPTIMATRIX v2.0 for the primary determinants thought to affect the reproducibility of micro-Matrix-based experiments where it's employed as an *ex vivo* human distal colon model, particularly in terms of occurrence of microbial blooms. These are namely: the faecal inoculum source, the type of FFM used, the duration the experiment is conducted for, and the systemic features unique to the micro-Matrix bioreactor platform. To this end, we evaluated the changes brought about to the faecal gut microbial community sourced from various individual (and pooled) donor samples throughout the duration of the experiment, in both Duran bottle and micro-Matrix bioreactor platforms and in all FFMs using both relative and absolute abundance metrics derived from 16S rRNA-based amplicon sequencing and flow cytometric methods respectively. Importantly, the absolute abundance metrics are generated by merging the relative abundance information from sequencing with the cell count/ml values from flow cytometry, that finally provides us with the actual cells per taxa per mL as described in our previous study [1]. This provides us a means to perform secondary validation of our findings, where microbial communities can be differentiated beyond their compostional relativities. This is particularly useful for monitoring taxa growth over time. For example, a particular taxa might grow in numbers but can maintain the same proportions in the microbiome, and this growth will therefore not be reflected in relative abundance metrics from 16S rRNA-based sequencing but will be captured in absolute abundance metrics. However, since these are not relative abundances, while making comparisons between samples, the total abundance must be considered carefully when making biological interpretations. Our findings from these validation experiments are described below in brief, with these validations being used to provide users with the standardised OPTIMATRIX v2.0 methodology, as described in the previous section. A schematic representation of the pipeline followed in this study is provided in Fig. 1. This was a single replicate experiment with combinations of timepoints, devices, faecal fermentation media, and faecal donor sources represented by one sample (Supplementary Table S1 and S2).

### Faecal fermentation media composition is one of the primary determinants driving the development of microbial blooms in *ex vivo* distal colon model experiments using the micro-Matrix platform

Until now, nine studies have been published where Fooks & Gibson media (FGM) has been used as the media of choice for batch fermentation experiments in the micro-Matrix bioreactor system used as an *ex vivo* model of the human distal colon [1,[8], [9], [10], [11], [12], [13], [14], [15]]. Some of these studies and others (data not shown) reported microbial blooms with the FGM media. Indeed, we previously reported partial optimisation of methods to mitigate *E. coli* blooms in *ex vivo* human distal colon model experiments conducted using the micro-Matrix platform with the use of the Fooks & Gibson Faecal Fermentation Media (referred to as FGM hereafter) [1,4]. While our optimised methods resulted in partial attenuation of such blooms, here we have followed up with further investigations by testing four different FFM compositions, in order to further mitigate such microbial blooms and identify the media which is most effective in this regard. While *E. coli* was the primary contributor to blooms in our previous investigations, we occasionally found that microbial blooms were also caused by *Ent. faecalis* and *Klebsiella michiganensis*. The phenomenon of such microbial blooms concurrently results in a significant loss of microbial diversity when compared to the starting (zero hour or T0) gut microbiota profile. In such experiments, the microbial blooms would typically involve between 1 and 3 taxa (genera or species) of microbes constituting a large percentage of the relative abundance. Since the majority of such microbial blooms involved coliforms such as *E. coli* and *K. michiganensis*, we hypothesised that such microorganisms are relatively fast-growing and require simple nutrients, as opposed to perhaps some of the other relatively fastidious gut microorganisms which may require the presence of more complex and/or specific nutrients, and the presence of strict anaerobic conditions similar to the human gut. Furthermore, any such fastidious gut microbes are likely to take comparatively longer to grow than fast-growing, metabolically versatile microorganisms such as *E. coli*, which would most likely give the latter a competitive advantage, ultimately resulting in such microbial blooms.

To investigate the impact of the FFM on maintenance of the faecal gut microbiota diversity and causing/mitigating microbial blooms in the micro-Matrix bioreactor platform, we tested four different FFM compositions (Table 1).

(i). Fooks & Gibson medium as described in our previous study [1,4].
(ii). Fooks & Gibson supplemented with 2.5 % w/v fructooligosaccharide (FOS) (hereafter referred to as FGMFOS in this study). The addition of FOS to the FGM was precipitated by the observation in previous experiments with the micro-Matrix that there was a noticeable drop in the *Bifidobacterium* populations [1]. FOS, known to elicit a bifidogenic effect in the gut, was added to the FGM to see if this modification enabled the FGM to maintain the *Bifidobacterium* spp. population better in such colon model experiments [6]. If not, it may indicate that the FFM might be deficient in certain critical nutritional aspects that might allow fastidious gut microbes to grow comparatively better in other media.
(iii). A modified version of the media largely based on the composition described in MacFarlane et al. [5] (hereafter designated at MCF in this study).
(iv). A modified version of a media largely based on the composition described in McDonald et al. [7] (hereafter designated as MCD in this study).

MCF and MCD were included as test media after a thorough literature search due to their richer composition, both in qualitative and quantitative terms, compared to FGM. Indeed, the MCF and MCD media that we used in this study contained 19 and 20 components respectively, in contrast to the 13 and 14 present in FGM and FGMFOS respectively (Table 1). We hypothesised that the richer composition of the MCF and MCD media, both of which contain inulin (with a known bifidogenic effect), casein (as an additional protein source), mucin (fed on by certain gut microbes), starch and pectin (with potentially prebiotic effects) would afford more fastidious microbes constituting the faecal microbiota an opportunity to grow and compete with faster growing microbes such as *Escherichia* spp., *Klebsiella* spp. and *Enterococcus* spp. Moreover, we surmised that some specific differences in the concentrations of certain salts and minerals between MCF, MCD and FGM may also contribute to attenuated microbial booms and consequent preservation of microbial diversity in MCF/MCD compared to FGM.

The effectiveness of the four media compositions was tested for different individual donor and pooled FSIs with micro-Matrix experiments for prolonged durations (up to 168 h; from T96 to T168, the micro-Matrix cassette was taken out of the micro-Matrix unit and incubated in an anaerobic hood). In our discussion of the effects of the different media compositions, we primarily focus on discussing the pooled FSI faecal fermentation samples, as these pooled FSI samples are traditionally used in such experiments. Individual donor FSI samples will be discussed wherever deemed applicable, with a detailed comparison made in a section below where we discuss the differential effect of faecal donor sources. Furthermore, with regards to discussion of the results in relation to the different FFMs, we will primarily concentrate on three key groups of gut microbes: (i) gut microbes contributing to microbial blooms such as the genera *Escherichia-Shigella, Klebsiella* and *Enterococcus*; (ii) gut microbes known to frequent foodstuffs and/or have potential probiotic properties such as representatives of the genera *Lactobacillus* and *Bifidobacterium*; and, (iii) gut microbes that are core resident microbes such as the genus *Bacteroides* or have been recently identified as being associated with health such as species from the genera *Faecalibacterium* and *Roseburia*. In this regard, we will validate our optimisations and recommendations as per relative and absolute abundance data generated for the *ex vivo* colon model experiment samples through 16S rRNA-based amplicon sequencing and flow cytometry respectively, as mentioned above. It is to be noted that for the purposes of the current work, the 16S rRNA amplicon sequences were classified using the SILVA SSU r138.2 database, which does not implement the updated classification for the genus *Lactobacillus* as yet [16,17]. Therefore, for example, species like *Lactobacillus plantarum* and *Lactobacillus casei*, which are now re-classified as *Lactiplantibacillus plantarum* and *Lacticaseibacillus casei*, among others, are included in the genus *Lactobacillus* for the present work.

**Fig. 2:**
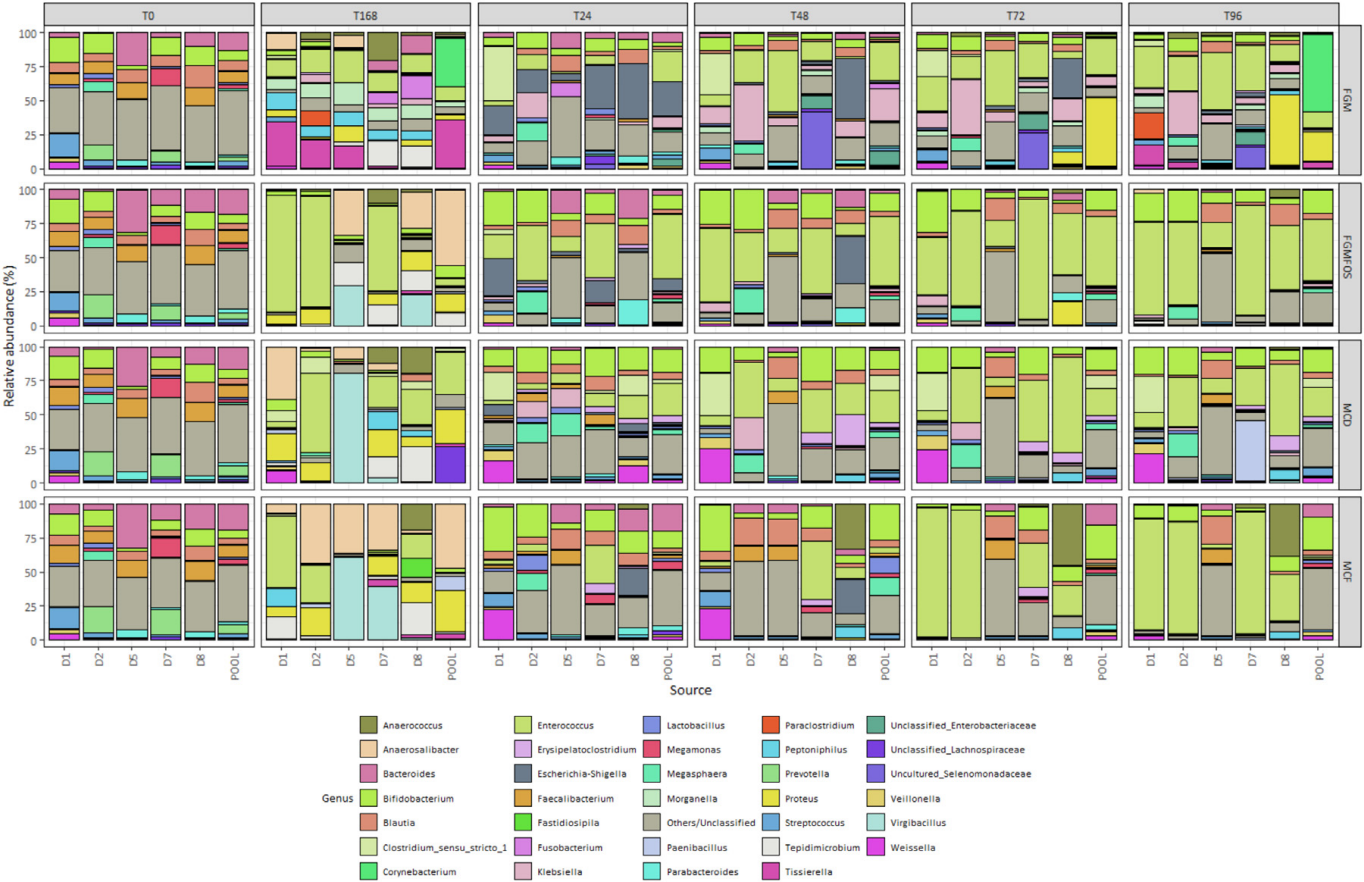
Taxonomic composition of fermentation experiments in the micro-Matrix bioreactor platform employed as an *ex vivo* distal colon model as revealed by 16S rRNA-based amplicon sequencing. The effects of different faecal fermentation medium, sampling time, systemic effects and faecal donor sources on the gut microbial community were determined using relative abundance metrics derived from 16S rRNA-based amplicon sequencing. Microbial compositions at the genus level for the gut microbial community in the faecal fermentation medium is shown here for T0 (start of experiment), T24 (24 hrs into the experiment), T48 (48 hrs into the experiment), T72 (72 hrs into the experiment), T96 (96 hrs into the experiment), T168 (end point of the experiment); MM: Micro-Matrix; D1, Donor 1; D2; Donor 2; D5, Donor 5; D7, Donor 7; D8, Donor 8 and POOL, Pooled FSI from eight donors. FGM, Fooks & Gibson media [1,4]; FGMFOS, Fooks & Gibson media supplemented with 2.5% w/v FOS; MCF, Modified media derived from MacFarlane et al. [5]; and MCD, Modified media derived from McDonald et al. [7]. Only genera present at a relative abundance of ≥ 10% in at least 1 sample are shown here (for more information please see Supplementary Table S1).

**Fig. 3:**
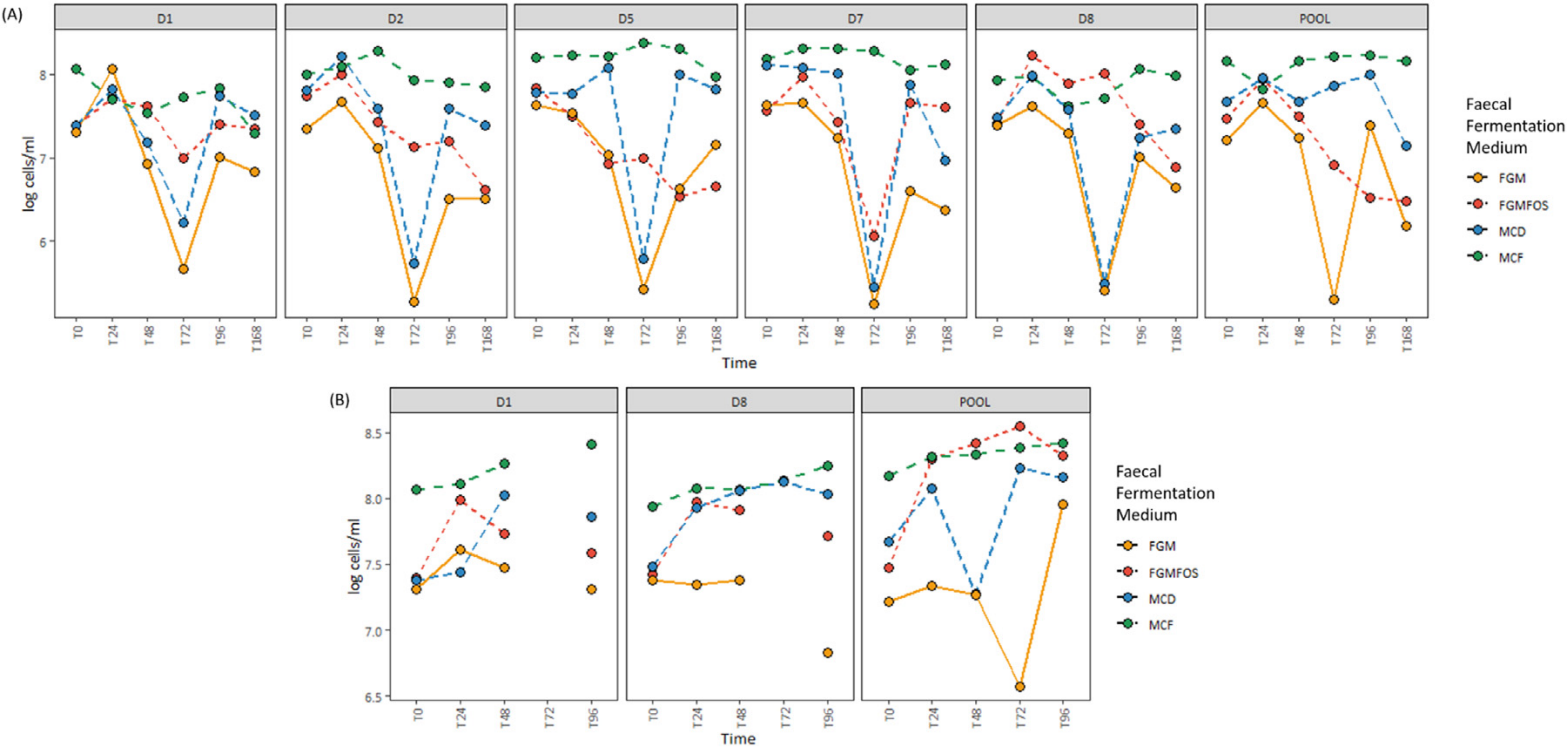
Total abundances for fermentation experiments in the micro-Matrix bioreactor platform and Duran bottles employed as an *ex vivo* distal colon model. Total abundances for faecal fermentation samples from the micro-Matrix as well as Duran bottles were quantified through a flow cytometric approach and expressed as the logarithm of cells per mL (LCM). Abbreviations: D1, Donor 1; D2; Donor 2; D5, Donor 5; D7, Donor 7; D8, Donor 8 and POOL, Pooled FSI from eight donors; FFM, Fooks & Gibson media [1,4]; FOS, Fooks & Gibson media supplemented with 2.5% w/v FOS; MCF, optimised media derived from MacFarlane et al. [5], MCD, optimised media derived from McDonald et al. [7]; T0 (start of experiment), T24 (24 hrs into the experiment), T48 (48 hrs into the experiment), T72 (72 hrs into the experiment), T96 (96 hrs into the experiment), T168 (end point of the experiment). For more information please refer to Supplementary Table S5.

First, we validated our results through determination of the total abundance of the colon model experiment samples, i.e. the total number of cells/ml of the fermentation. For pooled FSI colon model experiment samples, both MCF and MCD performed better than FGM and FGMFOS in maintaining the T0 levels of total abundance throughout the course of the experiment, with no drastic fluctuations (Fig. 2, Supplementary Table S5). Indeed, for MCF the T0 levels of total abundance were 8.16 log cells/ml (LCM) of fermentation with the greatest deviation from T0 being at T24 to 7.82 LCM (Fig. 3, Supplementary Table S5). Similarly for MCD, at T0 the total abundance was 7.67 LCM and the greatest deviation from T0 was observed at T168 with 7.14 LCM. It must be noted that between T96 and T168, there were no abiotic parameter controls of pH present as mentioned above. For FGM and FGMFOS however, there were major fluctuations in the total abundances across time points. For example, the total abundance for pooled FSI:FGM samples dropped to 5.3 LCM at T72 from 7.21 LCM at T0, while remaining at 6.18 LCM at T168 (Fig. 3, Supplementary Table S5). Similarly, for FGMFOS, the total abundance declined from 7.47 LCM at T0 to 6.91 LCM at T72, with the numbers gradually declining further to 6.52 LCM and 6.48 LCM for T96 and T168, respectively. Therefore, these observations suggested that MCD and MCF were superior to FGM and FGMFOS at maintaining the microbial numbers from faecal inocula in colon model experiments.

**Fig. 4:**
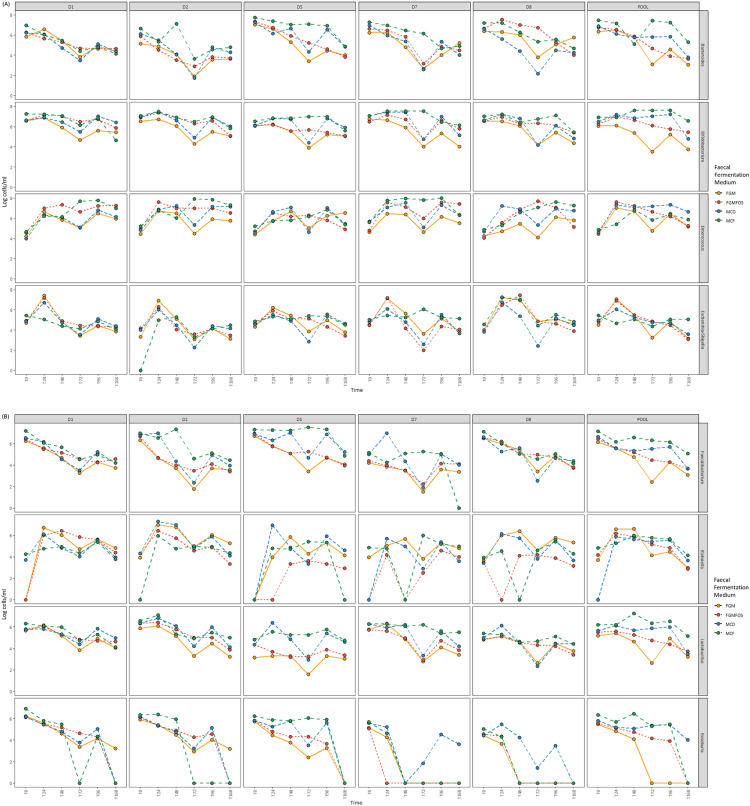
Absolute abundances for key genera in fermentation experiments in the micro-Matrix bioreactor platform employed as an *ex vivo* distal colon model. A flow cytometric approach was undertaken to quantify the total abundance in faecal fermentation samples from the micro-Matrix bioreactor platform employed as an *ex vivo* distal colon model. This data was merged with relative abundance data from 16S rRNA-based amplicon sequencing to obtain absolute abundances for key gut microbial genera of interest. Absolute abundances are expressed as the logarithm of cells per mL (LCM) of the faecal fermentation sample. (A) Absolute abundances for the genera *Bacteroides, Bifidobacterium, Enterococcus* and *Escherichia-Shigella.* (B) Absolute abundances for the genera *Faecalibacterium, Klebsiella, Lactobacillus* and *Roseburia*. Abbreviations: D1, Donor 1; D2; Donor 2; D5, Donor 5; D7, Donor 7; D8, Donor 8 and POOL, Pooled FSI from eight donors; FFM, Fooks & Gibson media [1,4]; FOS, Fooks & Gibson media supplemented with 2.5% w/v FOS; MCF, optimised media derived from MacFarlane et al. [5], MCD, optimised media derived from McDonald et al. [7]; T0 (start of experiment), T24 (24 hrs into the experiment), T48 (48 hrs into the experiment), T72 (72 hrs into the experiment), T96 (96 hrs into the experiment), T168 (end point of the experiment). For more information please refer to Supplementary Table S3.

When considering the taxa that are known to contribute to microbial blooms in micro-Matrix experiments, such as *Escherichia-Shigella, Klebsiella* and *Enterococcus,* MCD and MCF were found to be more efficient than FGM or FGMFOS at maintaining these genera at baseline proportions, i.e. at T0, for pooled FSI faecal fermentations. Briefly, at T0, the relative abundances for these genera for pooled samples in FGM were 0.19 %, 0.03 % and 0.16 % respectively with the relative abundances for FGMFOS being 0.22 %, 0.04 % and 0.09 % respectively (Fig. 2, Supplementary Table S1). The relative abundances for the same genera at T24 were 25 %, 8 % and 22 % for FGM, and 9 %, 1.6 % and 47 % respectively. In contrast, while the relative abundances of *Escherichia-Shigella, Klebsiella* and *Enterococcus* of MCD and MCF are comparable to those for FGM and FGMFOS at T0 (i.e. all <1 %), the relative abundances of these key genera implicated in microbial blooms is much lower at T24 for these FFM. Indeed at T24, with the exception of *Enterococcus* for MCD (23.4 %), both FFM attenuated the growth of these genera close to baseline levels (i.e. <1 %) (Fig. 2, Supplementary Table S1). At later time points such as T72 and T96 however, MCD does not perform as well compared to FGM and FGMFOS as MCF. For example, for pooled samples at T72, *Enterococcus* has a relative abundance of 26 %, 50 % and 20.2 % in FGM, FGMFOS and MCD, whereas for MCF it is only 0.42 %. Similarly, for pooled FSI samples at T96, FGM, FGMFOS and MCD show enrichment of *Enterococcus* with relative abundances of 11 %, 44 % and 21.4 % respectively, with MCF at 1.35 % (Fig. 2, Supplementary Table S1). Observations from 16S rRNA-based relative abundances were relatively consistent with absolute abundance metrics as well (Fig. 4, Supplementary Table S3). For example, for *Escherichia-Shigella*, the pooled FSI:FGM sample had an absolute abundance of 4.5 LCM at T0 and 7 LCM at T24, whereas for MCF, the corresponding values were 5.4 and 4.6 LCM respectively (Fig. 4, Supplementary Table S3). At T72, there was a drop in the absolute abundance of *Escherichia-Shigella* for pooled FSI:FGM samples due to the low total abundance numbers. In other time points such as T48 and T72, all other pooled FSI:FFM samples showed similar absolute abundances (Figs. 3, 4, Supplementary Table S3). Importantly, MCF was able to maintain the genus close to baseline absolute abundance levels throughout the duration of the experiment. For *Klebsiella*, we can observe variations between time points, particularly for FGM, but MCF and MCD largely maintain the genus close to baseline levels throughout the duration of the experiment. With regards to *Enterococcus*, the absolute abundance patterns showed how it grew in the pooled FSI:MCF samples over time as the total abundance of these samples increased, however, it still stayed below the absolute abundances of the genus at most time points for other FFM (Figs. 3, 4, Supplementary Table S3).

The second group of gut microbes we considered were the genera *Bifidobacterium* and *Lactobacillus*, which are commonly prevalent in various foods including fermented foods with some strains from these genera being classified as probiotics that can potentially colonise the gut [18]. Additionally, these genera are often important microbial proxies in gut microbiota studies that can indicate the impact of a specific intervention. For *Bifidobacterium*, the relative abundance at baseline (T0) for pooled samples was ∼5-6 % relative abundance, with these values increasing to 10-12 % for FGMFOS, MCD and MCF at T24 (Fig. 2, Supplementary Table S1). While FGM fails to maintain *Bifidobacterium* levels close to the relative abundance observed at baseline across all time points (<2 %), FGMFOS and MCD maintain the genus between 10 and 16 % from T24-T96, which although higher than T0 in pooled donor samples, is comparable to *Bifidobacterium* relative abundances in most original individual donor samples (Fig. 2, Supplementary Table S1). MCF, however, shows an increase in the levels of *Bifidobacterium* to a relative abundance of ∼25 % from T48-T96 with 12 % relative abundance at T24 for pooled donor samples. These observations are largely consistent with observations from absolute abundance metrics as well, where *Bifidobacterium* levels fall from 6 LCM at T0 for pooled FSI:FGM samples to 3.49 LCM at T72 and 5.19 LCM at T96, while for MCF, the *Bifidobacterium* levels rise from 6.91 LCM at T0 to 7.61 LCM at both T72 and T96 (Fig. 4, Supplementary Table S3). *Lactobacillus* spp. were present at T0 for pooled donor samples at a relative abundance ∼1 % and most FFM were able to maintain the genus at this level throughout the course of the experiment if only the relative abundance data was considered (Fig. 2, Supplementary Table S1). However, when considered in conjunction with the absolute abundance metrics, it's apparent that MCF performs much better than other FFM at maintaining *Lactobacillus* populations across all time points. As MCF performs better at maintaining/growing the gut microbiota in the fermentation experiments, it maintains *Lactobacillus* from 6.2 LCM at T0 for pooled samples through a range of 6.2-6.5 LCM across T24-T96 while maintaining their relative proportion in the faecal fermentation microbiota (Figs. 2, 4, Supplementary Tables S1, S3). In contrast, *Lactobacillus* in FGM for pooled samples dwindled in numbers from 5.19 LCM at T0 to 2.65 LCM at T72 and 4.91 LCM at T96, even though its population remained similarly proportional in the microbiota as at baseline levels (Figs. 2, 4, Supplementary Tables S1, S3). This was an important instance where the combinatorial approach of using relative and absolute abundance values gave us further insights compared to those possible only through the former. It is therefore advised to pursue such combinatorial approaches where possible, which in turn leads to more accurate inferences from a study.

The final group of key genera we looked at were *Bacteroides*, a core genus in the gut microbiota, as well as some genera that have more recently been studied due to their associations with aspects of health, i.e. *Faecalibacterium* and *Roseburia* [19]. We did not evaluate the important health associated gut genus *Akkermansia* as the primary individual donor samples did not have a high enough population of the genus (Supplementary Table S1). For *Faecalibacterium*, the relative abundances at baseline for all pooled FSI:FFM combinations were ∼8-9 % (Fig. 2, Supplementary Table S1). However, none of the FFM compositions were particularly good at maintaining these levels of *Faecalibacterium* throughout the course of the experiment. The relative abundances were usually <1 % for most time points for all FFM compositions with MCF supporting a relative abundance of 2.2 % and 1.29 % at T24 and T72 respectively (Fig. 2, Supplementary Table S1). Absolute abundance values for *Faecalibacterium* also show a declining trend for all FFMs from T0 through T168. However, MCF was the best at resisting the sharp decline in absolute population for *Faecalibacterium* in the fermentations, as was observed for FGM and FGMFOS across all time points (Fig. 4, Supplementary Table S3). *Roseburia* were identified at low relative abundances in the baseline pooled samples for all FFM (1-2 %). In terms of relative abundance, only MCF at T48 showed a comparable value similar to T0 (1.97 %); at all other time points and for all pooled FSI:FFM combinations, the relative abundances for *Roseburia* was less than 0.5 % (Fig. 2, Supplementary Table S1). Absolute abundance results for *Roseburia* were similar to those observed for *Faecalibacterium* in that there was a general decline among all FFM across time points with sharp declines in abundance seen beyond T48 for FGM and for FGMFOS after T96 (Fig. 4, Supplementary Table S3). MCF again was the best at resisting this gradual decline among the FFM, maintaining absolute abundance values close to baseline at least until T48 (T0: 6.32 LCM; T24: 5.68 LCM; T48: 6.46 LCM) (Fig. 4, Supplementary Table S3). Finally, *Bacteroides*, a core and usually abundant genus in a healthy gut microbiota was observed at relative abundances of 13-19 % among all pooled FSI:FFM combinations at the baseline. Moving through the time points for pooled FSI faecal fermentation samples, only MCF was able to maintain a comparable relative abundance ranging between 9 and 20 % with an aberrant relative abundance of 0.08 % at T48 (Fig. 2, Supplementary Table S1). For all other time points and FFMs, *Bacteroides* relative abundance was below 3 %, which is not representative of the gut microbiota. When looking at the absolute abundance, we can see that for all FFM except for MCF, there is a gradual and sometimes sharp decline in the abundance of *Bacteroides* as we move through T0 to T168 (Fig. 4, Supplementary Table S3). For MCF, the absolute abundance of *Bacteroides* remains at baseline levels and after T72 increases, indicating proper maintenance across all time points. The dip in absolute abundance for the genus at T48 can be considered an aberration given the trend is of a gradual upswing in absolute abundance for the genus in MCF from T0 to T168 (Fig. 4, Supplementary Table S3). Therefore, our studies suggested that while MCF was adequate in maintaining *Bacteroides* during faecal fermentations in the micro-Matrix bioreactor platform, it was not as efficient for *Faecalibacterium* and *Roseburia*, although it still performed markedly better than the other FFM. This may be an avenue that requires further investigation and optimisation in future studies.

While this was a single-replicate design experiment that precluded the analysis of diversity metrics, we looked into the number of ‘observed features’ (used synonymously with amplicon sequencing variants or ASVs) detected for different FFM combinations at different time points (in a rarefied dataset of 28,460 sequences) to gain a deeper understanding of our experiments. When looking at the observed features for pooled FSI samples in micro-Matrix experiments, it was apparent that MCD and MCF performed better than FGM across all time points (Table 2, Supplementary Table S6, S7, and Supplementary Figure S2). While the observed features for FGM dropped from 672 at T0 to 108 at T96, they were maintained between 343-380 and 189-452 for MCD and MCF respectively from T0 to T96 (Table 2). In single donor FSI samples, however, the changes were less drastic due to the much lower microbial diversity of native single donor samples compared to pooled ones with FGM performing similar to MCF/MCD. For Donor 5, for example, the number of observed features for FGM fell from 326 at T0 to 256 at T96. During the same time points, observed features in MCD were 337 and 277 respectively and 313 and 262 respectively for MCF (Supplementary Table S7). It's noticeable that the starting microbial diversity of individual donor samples is much lower compared to pooled ones with FGM performing equally well or slightly worse than MCF/MCD in maintaining microbial diversity through the experiment's duration. This not only indicates that pooling does indeed create a much more diverse, artificial, ‘N'th’ microbiome but also that while FGM might be adequate for maintaining low diversity gut microbiota, MCD and MCF may perform better at maintaining moderately diverse gut microbiotas. We further looked into the shared observed features and unique genera for each FFM combination at different time points that furthered this inference. Indeed, for pooled FSI micro-Matrix samples between T24 and T96, only 3-5 unique genera were supported by FGM while MCD and MCF supported 6-16 and 3-10, indicating the latter's ability to maintain a greater diversity of gut microbes (Table 2). Additionally, across time points, FGM shares few ASVs with MCD and MCF with greater overlaps within the latter two, indicating potentially a different subset of the gut microbiota supported by MCD and MCF. For example, using rarefied data at T72, only 7 and 10 ASVs overlap between FGM:MCD and FGM:MCF respectively, with overlaps of MCD:MCF noted at 60 (Supplementary Figure S2). Interestingly, across most time points, FGMFOS, MCD and MCF show significant trilateral overlap of ASVs (T72: 55; T96: 100). Additionally, MCD and MCF usually maintained a much higher number of non-shared ASVs across time points compared to FGMFOS (T48: FGMFOS: 49, MCD: 114, MCF: 81; T72: FGMFOS: 48, MCD: 106, MCF: 147; T96: FGMFOS: 54, MCD: 110, MCF: 58) (Supplementary Figure S2). These two observations taken together may indicate a common subset of gut microbes supported through the bifidogenic and prebiotic activity accorded by these FFM and also that MCF and MCD potentially support a broader range of gut microbes beyond those that are bifidogenic in nature. We excluded results from T168 for reporting microbial diversity as the discontinuity in the experiment and the consequent depletion of nutrients caused a drastic reduction in microbial diversity across all FFM irrespective of the donor source and can therefore be misleading when interpreting (Supplementary Table S6, S7).

**Table 2:** Observed features (amplified sequencing variants) in rarefied data from Micro-Matrix (MM) for pooled donor frozen standardised inoculum samples. Detected microbial blooms, causal microorganisms and unique microbes at the genus level for diferent faecal fermentation media and time points are indicated. Detection of unique genera was carried out with native taxonomic compositional output while microbial blooms were detected in data filtered by taxa present in at least one sample at a relative abundance of ≥ 1%. See Supplementary Tables S6 and S7 and Supplementary Figure S2 for further details.

Time points#	Faecal fermentation medium*	micro-Matrix pooled FSI samples (MM_POOL)		Number of unique genera$
		Observed features	Number of genera blooming (>20% Relative abundance)	
T0	FGM	672	0	-
	FGMFOS	698	0	-
	MCD	692	0	-
	MCF	673	0	-
T24	FGM	382	2 (*Enterococcus; Escherichia-Shigella*)	3 (*Woesearchaeales, Corynebacterium, Atopobium*)
	FGMFOS	406	1 (*Enterococcus*)	6 (*Sanguibacteroides, Paenibacillus, UCG-008, DTU089, Peptostreptococcus, Anaerovibrio*)
	MCD	343	1 (*Enterococcus*)	6 (*Gordonibacter, Rikenella, Rikenellaceae RC9 gut group, Clostridia vadinBB60 group, Clostridium sensu stricto 3, Paeniclostridium*)
	MCF	452	1 (*Enterococcus*)	9 (*Barnesiella, Catenibacillus, Hungatella, Eubacterium oxidoreducens group, Eubacterium xylanophilum group, UCG-009, Oscillospira, Family XIII AD3011 group, Mogibacterium*)
T48	FGM	275	2 (*Enterococcus; Klebsiella*)	5 (*Corynebacterium, Anaerostignum, Eisenbergiella, Phocea, Gallicola*)
	FGMFOS	289	1 (*Enterococcus*)	11 (*Methanobrevibacter, Libanicoccus, Muribaculaceae, Anaerofustis, Defluviitaleaceae UCG-011, Intestinimonas, Paludicola, Peptostreptococcus, Anaerosalibacter, Anaerovibrio, Oxalobacter*)
	MCD	371	1 (*Enterococcus*)	9 (*Odoribacter, Coprobacillus, Gemella, Eubacterium xylanophilum group, Flavonifractor, Oscillibacter, DTU089, Negativibacillus, Parasutterella*)
	MCF	189	1 (*Bifidobacterium*)	3 (*Paenibacillus, Lachnospiraceae UCG-001, Tepidimicrobium*)
T72	FGM	194	2 (*Enterococcus; Proteus*)	2 (*Corynebacterium, Virgibacillus*),
	FGMFOS	273	1 (*Enterococcus*)	6 (*Deep Sea Euryarchaeotic Group, Methanosphaera, Lactococcus, Paenibacillus, Anaerosalibacter, Anaerovibrio*)
	MCD	380	1 (*Enterococcus*)	16 (*Atopobium, Raoultibacter, Barnesiella, Prevotella, Facklamia, Leuconostoc, GCA-900066575, Lachnospiraceae UCG-008, Eubacterium ruminantium group, Intestinimonas, DTU089, Pygmaiobacter, Terrisporobacter, DTU014, Oxalobacter, Haemophilus*)
	MCF	412	1 (*Bifidobacterium*)	10 (*Odoribacter, Muribaculaceae, Dielma, CAG-56, Lachnospiraceae UCG-010, Eubacterium xylanophilum group, UCG-009, Oscillibacter, Mogibacterium, Parasutterella*)
T96	FGM	108	2 (*Corynebacterium; Proteus*)	5 (*Prevotellaceae NK3B31 group, Rikenellaceae RC9 gut group, Paraclostridium, Gallicola, Fusobacterium*)
	FGMFOS	280	1 (*Enterococcus*)	5 (*Howardella, Oribacterium, UCG-003, Family XIII AD3011 group, Terrisporobacter*)
	MCD	363	1 (*Enterococcus*)	12 (*Methanosphaera, Butyricimonas, Muribaculaceae, Coprobacillus, Facklamia, Leuconostoc, GCA-900066575, DTU089, Paludicola, Pygmaiobacter, Family XIII UCG-001, Citrobacter*)
	MCF	271	1 (*Bifidobacterium*)	5 (*Clostridia vadinBB60 group, Paeniclostridium, Allisonella, Negativicoccus, Parasutterella*)
T168	FGM	137	2 (*Corynebacterium; Tissierella*)	-
	FGMFOS	146	1 (*Anaerosalibacter*)	-
	MCD	86	2 (*Enterococcus; Proteus*)	-
	MCF	102	2 (*Enterococcus; Proteus*)	-

# T0: zero hours; T24: 24 hours; T48: 48 hours; T72: 72 hours; T96: 96 hours, and T168: 168 hours after start of faecal fermentation

⁎ FGM: Fooks and Gibson medium; FGMFOS: FGM supplemented with 2.5% (w/v) fructooligosaccharides; MCD: modified McDonalds Medium; MCF: modified MacFarlane medium

$ Number of unique genera in for the medium compared to other media at that particular time point (done only for T24, T48, T72, and T96)

Overall, all evidence taken together, MCF was clearly the best suited FFM to not only mitigate microbial blooms arising from *Escherichia-Shigella, Klebsiella* and *Enterococcus*, but also performed better in maintaining core gut microbes and other microbes of interest such as *Lactobacillus, Bifidobacterium, Bacteroides* and *Faecalibacterium,* close to baseline levels, with overall growth of the faecal microbiota observed. Both MCF and MCD were also potentially better at maintaining a greater diversity of gut microbes compared to FGM including uniquely supported, non-bifidogenic, obligately anaerobic gut microbial taxa. While we have mostly discussed pooled faecal fermentation samples here, this pattern largely holds up for individual donor samples as well, with the best results observed at T72-T96. Moving forward, we therefore recommend the use of MCF in lieu of other FFM for experiments in the micro-Matrix bioreactor platform employed as an *ex vivo* distal colon model (see OPTIMATRIX v2.0 above and below for further information).

### Prolonged durations of fermentation in *ex vivo* distal colon model experiments using the micro-Matrix bioreactor platform might provide more consistent results

Previous studies involving the use of the micro-Matrix bioreactor platform have typically been conducted for relatively short durations such as 12 h [[9], [10], [11]], 24 h [8,[12], [13], [14], [15]] and 48 h [1]. Here, we aimed to evaluate whether there was a temporal effect on the appearance of microbial blooms and loss of microbial diversity in colon model experiments. To this end, we conducted such colon model experiments in the micro-Matrix system up to 96 h (T96) with a final sampling at 168 h as mentioned above. Importantly, while the shorter duration of previous micro-Matrix-based colonic studies obviated the need to replenish the FFM to maintain a constant supply of nutrients for the faecal microbiota, a much longer experimental duration of 96 h would most likely result in utilisation and consequent depletion of all the nutrients present in the FFM, as well as a consequential build-up of toxic waste. To avoid such an eventuality, we removed aliquots of the FFM:FSI mix every 24 h for sampling purposes, while simultaneously replenishing spent nutrients by replacing the volume of media removed with fresh FFM. By doing so, we loosely mimicked semi-continuous models of fermentations where the same is achieved through an arrangement of pumps and tubes. Our hypothesis was that prolonged fermentation durations would afford the faecal microbiota an adaptation/stabilisation period akin to the SHIME and TIM-2 systems, and would consequently mitigate microbial blooms, especially in the latter time points of 72 h (T72) and 96 h (T96). Similar to the previous section, we will restrict ourselves to discussing the observations from only pooled FSI faecal fermentation samples with discussions on individual donor FSI faecal fermentation samples delegated to the next section.

Consistent with the results from our previous study [1], the phenomenon of *E. coli* blooms is somewhat attenuated at prolonged time points (such as at 48 hours compared to 24 h), with 24 h after the start of the colon model experiment being where *E. coli* blooms are most pronounced. For example, for the pooled FSI in FGM and FGMFOS, the relative abundance of the genus *Escherichia-Shigella* at T24 was 25 % and 9 % respectively (0.19 % and 0.22 % at T0 respectively) with the corresponding values being 1.7 % and 0.9 % at T48, and 0.88 % and 0.84 % at T72 (Fig. 2, Supplementary Table S1). *Escherichia-Shigella* levels were maintained below 1 % relative abundance in the pooled FSI faecal fermentation samples for MCD and MCF (Fig. 2, Supplementary Table S1). Indeed, at least for the genus of *Escherichia-Shigella*, the blooms can be conclusively inferred as a time point issue as all FFM are able to maintain the absolute abundance of this genus comparable to at T0 (∼4-5 %) at time points beyond T48 (∼3-5 %) (Fig. 4, Supplementary Table S3).

Another genus of interest vis-à-vis microbial blooms, *Enterococcus*, contributed to microbial blooms at T24, T48 and T72 in FGMFOS with relative abundances of 47.4 %, 50.71 % and 50.94 % respectively in pooled FSI faecal fermentation samples (Fig. 2, Supplementary Table S1). Compared to T0, both MCD and FGM also showed significant enrichment of *Enterococcus* across all time points, with T0 levels being ∼0.1 % relative abundance for both, while at all other time points the relative abundance ranged between 10 and 31 % (Fig. 2, Supplementary Table S1). None of the time points were therefore suitable in terms of attenuating *Enterococcus* microbial blooms; the only way to mitigate this is to resort to the use of MCF, which controlled the *Enterococcus* population below 4.6 % across all time points (Fig. 2, Supplementary Table S1).

*Klebsiella*, another genus occasionally observed in microbial blooms in micro-Matrix experiments, varied in relative abundance according to time points. For example, while MCD and MCF were overall better at maintaining lower levels of the genus in faecal fermentation samples from all time points for pooled FSI, FGM exhibited significant enrichment of the genus at T24-T72 with relative abundances of ∼8-24 % compared to ∼0.05 at T0 but normalised at T96 (Fig. 2, Supplementary Table S1). Similar to *Escherichia-Shigella*, this may be indicative of any particular deficiencies in the medium itself (as FGMFOS does not show the same patterns) or reflective of the specific microbial succession dynamics for this FFM.

For MCF, microbial blooms for *Bifidobacterium* were apparent at most time points in pooled FSI faecal fermentation samples. Indeed, while at T0 the *Bifidobacterium* relative abundance is ∼6 % it increases to 12 % at T24 and thereafter to ∼25 % at other time points, except at T168 (Fig. 2, Supplementary Table S1). At most time points, FGMFOS and MCD maintain *Bifidobacterium* at a relative abundance of ∼5-15 % with FGM failing to do so across all time points (Fig. 2, Supplementary Table S1). Interestingly, when the same observations are made for absolute abundances, at T72 and T96, the differences between the *Bifidobacterium* abundances for MCD and MCF are not as drastic. Indeed, at T72 and T96, the absolute abundance of *Bifidobacterium* are comparable for both media. Although higher compared to T0, the absolute abundance for *Bifidobacterium* in MCD and MCF indicated a healthy maintenance of this important gut genus compared to FGM, where there was a 10-100-fold reduction at T72 and T96 (vs T0) (Supplementary Table S3).

Interestingly, at T72 there is an observable drop in the total abundance and as a consequence, the absolute abundance of key genera in FGM and FGMFOS (Fig. 3, Fig. 4, Supplementary Table S3, S5). Indeed, the total abundance for FGM and FGMFOS in pooled FSI faecal fermentation samples at T48 is 7.23 and 7.47 LCM while at T72 it is 5.3 LCM and 6.9 LCM. However, a general revival of the microbial abundance is evident at T96 (Supplementary Table S5). Given that this pattern is observed for simulated *ex vivo* distal colon model experiments conducted in the Duran bottles as well, although only for FGM and not FGMFOS, it is an interesting open question as to why this happens. However, it is clearly an artefact of FGM. All observations taken together, as discussed above in brief, T96 seems to be a preferable sampling time point for such experiments, particularly when using FGM or FGMFOS.

### Pooled donor FSI performs better or equally well compared to individual donor FSIs in *ex vivo* colon model experiments conducted using the micro-Matrix bioreactor platform

Currently, there are divergent views with regards to the feasibility of using pooled faecal samples or faecal samples derived from individual donors for the purposes of *ex vivo* colon model experiments [20]. In the present work, we aimed to investigate whether microbial blooms were more likely or less likely when individual donor FSI was used as compared to a pooled donor FSI, when using the micro-Matrix bioreactor system. We generated pooled donor FSI from a pooled faecal mix of faeces from 8 volunteers, and generated separate individual donor FSI batches from 5 donors. We were unable to generate the 3 remaining individual donor FSI batches due to relatively small quantities of faeces donated by the 3 respective volunteers; adding the individual donor faecal samples to the pooled FSI was therefore prioritised over preparation of individual donor FSI. For the purposes of this section, we will restrict our observations and discussions to primarily those made in T72 and T96 (the preferred time points as established above) with references to other time points as necessary.

To understand the effects of different donor faecal sources on microbial blooms, we primarily concentrated on the key microbial genera usually implicated in the same, namely, *Escherichia-Shigella, Klebsiella* and *Enterococcus*. At T72 and T96, for all combinations of FFM and donor sources, the relative abundance of *Escherichia-Shigella* remained similar to those found at T0 (<1 %), with the exception of the donor 8:FGM sample (28.5 %) (Fig. 2, Supplementary Table S1). We observe something similar with *Klebsiella*, with its relative abundances being similar to T0 at T72 and T96 for most donor FSI:media combinations, with the exceptions of donor2:FGM and donor8:FGM at T72 (40.5 % and 16 % relative abundances) and again donor2:FGM at T96 (∼31 % relative abundance) (Fig. 2, Supplementary Table S1). These observations in individual donor samples are consistent with our previous observations for pooled faecal samples above, in that the microbial blooms for *Escherichia-Shigella* and *Klebsiella* are most likely a temporal product with later time points showing relative abundances similar to T0. Interestingly, the common denominator in the samples showing microbial blooms for these two genera is FGM, which again indicates that FGM is probably not the best suited for micro-Matrix experiments compared to MCD or MCF, as mentioned before. Additionally, microbial blooms for *Escherichia-Shigella* and *Klebsiella* are seen for only donor 2 and 8, indicating that although the T0 relative abundance levels of these genera in these two donor samples were low, the overall unique gut microbiota composition may have made these two donor samples predisposed to such microbial blooms, at least for FGM.

Observations for *Enterococcus*, the third microbial genus usually implicated in microbial blooms, were quite different however. At T72, most individual donor as well as pooled donor:FFM combinations showed high relative abundances for *Enterococcus* (20-94 %) (Fig. 2, Supplementary Table S1). Some notable exceptions were pooled FSI:MCF (relative abundance of 0.42 %) and donor 5:MCF (0.62 %), which were comparable to T0 values. Similar observations were made at T96, where for almost all donor and FFM combinations, *Enterococcus* were enriched with relative abundances up to 81 % of the microbial community (Fig. 2, Supplementary Table S1). Again, the notable exception, which presented a relative abundance level somewhat close to T0 at T96 was the pooled FSI: MCF sample with an *Enterococcus* relative abundance of 1.35 % (Fig. 2, Supplementary Table S1). These observations for *Enterococcus* highlight that besides time point, the choice of donor sample as well as FFM in combination is an important consideration for extracting the most physiologically representative results from micro-Matrix-based simulated *ex vivo* distal colon experiments as the common denominators for T0-like results at T96 were the pooled FSI and MCF.

Besides *Escherichia-Shigella, Klebsiella* and *Enterococcus*, there were a few other genera that exhibited sporadic microbial blooms. For example, at T72, *Anaerococcus* contributed to a relative abundance of 45.32 % for donor 8 FSI:MCF, while *Proteus* and uncultured Selenomonadaceae bacterium were enriched in pooled FSI:FGM and donor 7 FSI:FGM respectively, with relative abundances of 50.2 % and 26.5 % respectively (Fig. 2, Supplementary Table S1). *Bacteroides* and *Bifidobacterium* were enriched in the pooled FSI:MCF samples at T72 at relative abundances of 15.7 % and 25.2 %, respectively. Although not exactly representative of the gut microbial community, these abundances were considered less of an issue compared to *Proteus* or *Anaerococcus* as *Bacteroides* and *Bifidobacterium* are normally found in discernible numbers in the gut microbiota with the T0 values of these genera varying between 3 and 18 % across all donors and pooled FSIs. Similar trends were observed for *Anaerococcus, Proteus* and uncultured Selenomonadaceae at T96 for the exact same FSI:FFM combinations as at T72 mentioned above with relative abundances of 38.5 %, 21.3 % and 16.16 % (Fig. 2, Supplementary Table S1). Additional microbial blooms were detected for *Clostridium_sensu_stricto_1* and *Weisella* for donor 1 FSI:MCD and *Corynebacterium* for pooled FSI:FGM at T96 with relative abundances of 26.8 %, 21.3 % and 56.72 % respectively (Fig. 2, Supplementary Table S1). These genera are usually present at low relative abundances in the gut (≤5 %; Fig. 2, Supplementary Table S1), therefore such microbial blooms can become an issue. This can however be avoided with the use of MCF at T96, which was not indicted in such microbial blooms, thus further highlighting the necessity of using the combined recommendations of time points, FFMs and faecal donor source when attempting to conduct a close-to-exact, physiologically relevant distal colon study using the micro-Matrix bioreactor platform.

Taken together, the observations made for individual donor FSI faecal fermentation samples against pooled FSI faecal fermentation samples indicate that predispositions for microbial blooms in the micro-Matrix bioreactor platform employed as an *ex vivo* distal colon model might be avoided by the use of pooled FSI in lieu of individual donor FSI. However, we have also noted that using a pooled FSI may not be enough to ensure best possible results, and a combination of optimal FFM and sampling time points need to be considered as well. Indeed, pooled FSI samples perform distinctly better in terms of absolute abundance across time points, particularly at T72 and T96, compared to individual donor samples but only for MCD and MCF FFM (Fig. 2, Supplementary Table S5). It is to be noted that since each bioreactor system is different, this is not necessarily an argument for the use of pooled FSIs in other *in vitro*/*ex vivo* distal colon models; indeed, each research question would merit careful deliberation on the best source of faecal microbial communities.

### Corresponding investigations using Duran glass bottles in anaerobic chambers for simulated *ex vivo* colon model experiments in lieu of the micro-Matrix bioreactor platform

The final determinant we sought to assess in terms of its effect on development of microbial blooms, maintenance of microbial diversity and reproducibility of results was the systemic peculiarities of the micro-Matrix bioreactor platform itself. In order to understand if such microbial blooms are associated solely with the micro-Matrix system or if they might occur in near identical batch fermentation experiments, we used Duran glass bottles incubated in anaerobic chambers to conduct similar experiments to those conducted in parallel in the micro-Matrix bioreactor platform as described above. Importantly, even though the experimental design for these experiments conducted in bottles was similar to those conducted in the micro-Matrix unit, there were some important differences namely, the lack of pH control throughout the 168 h fermentation period, the lack of replenishment of the relevant faecal fermentation media, and the absence of agitation conditions in these bottles, with fermentations instead being conducted under static conditions. Thus, it is plausible that the lack of pH control and associated decrease in pH due to the production of acids as a by-product of microbial fermentation would likely affect the microbial diversity within the bottles. More specifically, it is likely that the environmental conditions pertaining inside the Duran glass bottles during the 168 h batch fermentation likely favoured the growth of microbial species that are less sensitive to acids/acid-tolerant in lieu of acid-sensitive microbial species. Other than the aforementioned differences however, all other conditional parameters such as the composition of the four FFM media tested, dissolved oxygen, and temperature were identical to those in the micro-Matrix unit. Notably, due to limited amounts of faecal samples available to generate sufficient individual donor sample FSI, for the purposes of these proof-of-concept experiments conducted in Duran glass bottles, we opted to use only two batches of individual donor FSI (i.e. FSI donor 1 and FSI donor 8). Additionally, the same pooled FSI prepared for the micro-Matrix experiments was also employed for the studies using the Duran bottles. Finally, although the experiments in bottles were conducted for 168 h, as was the case for the micro-Matrix experiment, for the purposes of cost-efficiency, whilst endeavouring to gain as many insights as possible, we opted to sequence a subset of Duran bottle samples (T24, T48, T72 and T96). The T0 samples were not sequenced, as in theory, the T0 starting inocula and associated microbiome profiles would have been identical to the T0 samples which were sequenced from the micro-Matrix experiment, as the systemic differences accorded to the experimental setup by the latter would not have had an opportunity to impact the fermentations.

**Fig. 5:**
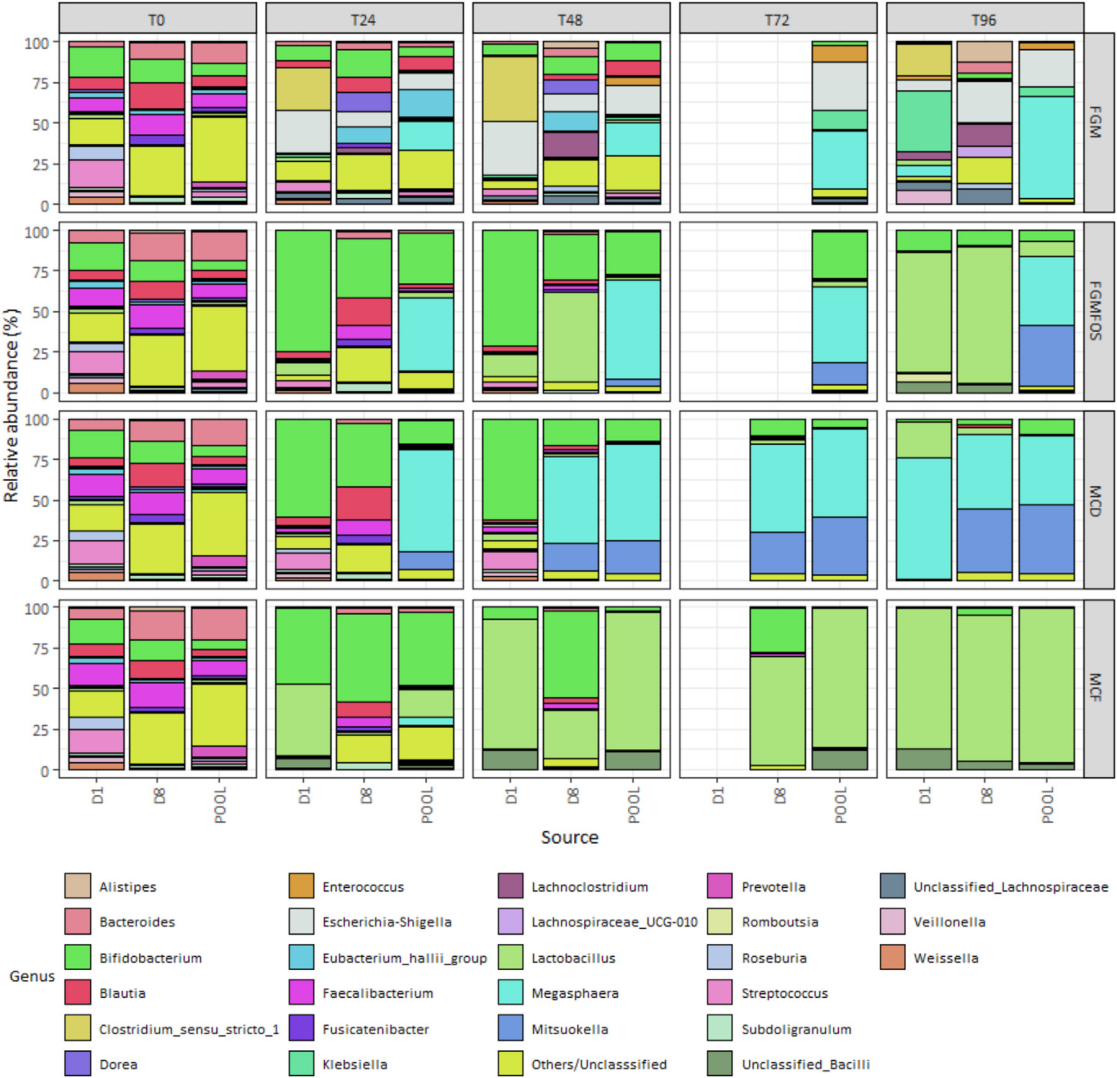
Taxonomic composition of fermentation experiments in Duran bottles employed as an *ex vivo* distal colon model as revealed by 16S rRNA-based amplicon sequencing. The effects of different faecal fermentation medium, sampling time, systemic effects and faecal donor sources on the gut microbial community were determined using relative abundance metrics derived from 16S rRNA-based amplicon sequencing. Microbial compositions at the genus level for the gut microbial community in the faecal fermentation medium is shown here for T0 (start of experiment), T24 (24 hrs into the experiment), T48 (48 hrs into the experiment), T72 (72 hrs into the experiment), and T96 (96 hrs into the experiment); BOT: Duran bottles; D1, Donor 1; D8, Donor 8 and POOL, Pooled FSI from eight donors. FGM, Fooks & Gibson media [1,4]; FGMFOS, Fooks & Gibson media supplemented with 2.5% w/v FOS; MCF, Modified media derived from MacFarlane et al. [5]; and MCD, Modified media derived from McDonald et al. [7]. T0 samples represented here were supplemented from the T0 16S rRNA amplicon sequencing data from the micro-Matrix samples (see main text for more information). Only genera present at a relative abundance of ≥ 5% in at least 1 sample are shown here (for more information see Supplementary Table S2). Due to a limited number of index combinations available for library preparations for sequencing, we opted to exclude preparing certain samples for sequencing at the T72 time point for these Bottle experiments, as they were of a lower priority compared to the above mentioned micro-Matrix samples.

In general, experiments carried out in the Duran bottles were found to be more susceptible to microbial blooms compared to counterpart experiments in the micro-Matrix bioreactor platform. For example, the pooled FSI faecal fermentation samples, which performed the best in the corresponding micro-Matrix experiments, showed high levels of microbial blooms compared to the micro-Matrix bioreactor platform. For example, *Lactobacillus* produced significant blooms in the pooled FSI-MCF samples in the Duran bottles with relative abundances of 16 %, 85 %, 85 % and 95 % at T24, T48, T72, and T96, respectively (Fig. 5, Supplementary Table S2). Such relative abundances where one taxa is completely taking over the fermentation has not been observed in the micro-Matrix. The favourability of *Lactobacillus* in such circumstances might be due to the growing acidity in the faecal fermentation over time that is tolerated by *Lactobacillus* but not by other gut microbial species. *Bifidobacterium*, which is well supported in MCF, was the most prominent genus in the pooled FSI-MCF sample with a relative abundance value of 44.6 % (Fig. 5, Supplementary Table S2). Notably, while elevated levels of *Bifidobacterium* were observed in some MCF time points for the corresponding micro-Matrix experiments (∼20-25 %), such high relative abundances were not. These observations are also supported by absolute abundance-based results from the same experiments for *Lactobacillus* and *Bifidobacterium* (Supplementary Figure S1, Supplementary Table S4). Importantly, while both *Lactobacillus* and *Bifidobacterium* are known to inhabit/colonise the gut, they are not representative of the major taxa found in the gut microbiome; microbial blooms contributed by these genera therefore compromises the faecal microbial community in a way that it stops being representative of the gut microbiota.

Interestingly, the microorganisms causing such blooms were sometimes ones that have not been encountered before as a causal microbe in micro-Matrix experiments. For example, at T24 for the pooled FSI faecal fermentation samples, *Meghasphaera* was the genus which dominated in Duran bottle experiments with a relative abundance of 17.3 %, 45.7 % and 62.6 % for FGM, FGMFOS and MCD, respectively (Fig. 5, Supplementary Table S2). At T48, *Meghasphaera* continued to contribute to microbial blooms with relative abundances of 20.9 %, 61.2 % and 60 % for FGM, FGMFOS and MCD respectively. In contrast, the corresponding numbers for *Megasphaera* from micro-Matrix experiments were 0.03 %, 0.003 % and 0.08 % relative abundance for FGM, FGMFOS and MCD respectively with other time points also showing near zero relative abundances. The genus *Mitsuokella* is another example of a unique microbe encountered as a contributor to microbial blooms that has not been previously encountered in micro-Matrix experiments. Indeed, in the pooled FSI-MCD faecal fermentation samples in Duran bottles, it contributes to a relative abundance of 20 % at T48, 35 % at T72, and 42 % at T96 (Fig. 5, Supplementary Table S2). *Mitsuokella* was also significantly enriched at T96 in the FGMFOS-pooled FSI faecal fermentation samples at T96 with a relative abundance of 37 %. Importantly, neither *Megaspaera* nor *Mitsuokella* are known as major members of the gut microbiota; microbial blooms derived from these genera therefore compromises the integrity of the faecal microbial community and the biological relevance of the experiment.

When looking at the microbial diversity of the Duran bottle samples across time points for pooled FSI samples, we notice that the ASVs reported for FGM are comparable to those reported in the micro-Matrix samples (Supplementary Table S6). However, the ASVs reported for MCD and MCF were much lower, not only compared to corresponding micro-Matrix samples but also to FGM samples in Duran bottles. For example, at T72 and T96, FGM samples had 222 and 112 observed features compared to 75 and 56 for MCD and 72 and 40 for MCF respectively. These patterns were also largely consistent in individual donor FSI samples across time points (Supplementary Table S7). These somewhat aberrant outcomes were probably due to a differential effect of the particular conditions of the Duran bottle-based setups (no pH control, no agitation, limitation of dissolved oxygen etc.) on specific components of MCD and MCF (such as minerals and prebiotics) that would contribute to them supporting a broad range of gut microbes compared to FGM, which is a far simpler media.

While only a brief discussion of our results from the Duran bottle-based faecal fermentations are presented here, it was quite clear that the lack of pH control in the bottles in contrast to the micro-Matrix system is crucial to maintain the faecal microbial community integrity during the fermentation. Without this pH control, the microbial community in the faecal fermentations rapidly changes into one favouring the growth of only a few taxa and becomes non-representative of the gut microbiota. We concluded therefore that the systemic features of the micro-Matrix were not a contributor to the microbial blooms observed in micro-Matrix based experiments, and on the contrary, they indeed helped maintain the integrity of the faecal/gut microbial community.

## Concluding remarks

Mitigation of microbial blooms and concomitant preservation of true gut microbiota diversity is an important step enabling improved accuracy of *ex vivo* distal colon model-based studies, which in turn allows us to bridge the gap between such investigations and potential downstream *in vivo* pre-clinical studies in animals (e.g., rodents, pigs) and human intervention (or clinical) trials. In the present study, we have gained some additional, critical insights into optimising the operational methodology for *ex vivo* human distal colon model experiments using the micro-Matrix bioreactor platform. Overall, we make three minimum recommendations: (1) We recommend users to use the modified MCF medium (described in Table 1) derived from the MacFarlane medium [5] and optimised for use here, as a FFM for mitigating any problems encountered with microbial blooms and concomitant loss of microbial diversity in such experiments; (2) We additionally recommend researchers continue using pooled FSI instead of individual donor faecal samples, as our findings here indicate that the latter is more prone to microbial blooms and a concomitant loss of microbial diversity; (3) We further recommend that users conduct micro-Matrix experiments for slightly prolonged durations (at least 72 hours and up to 120 hours when possible) instead of 24 hours, by regularly aliquoting and replenishing the fermentation matrix with fresh media. It is to be noted that these recommendations are to be used in combination and not in isolation, as that will lead to different consequences. By following the recommendations made here, researchers should be able to negate microbial blooms, preserve microbial diversity and improve reproducibility of their results while using the micro-Matrix bioreactor platform as an *ex vivo* distal colon model. Ultimately, once implemented, our recommendations should enable the micro-Matrix system employed as a distal colon model to become a more reliable predictor of the impact of different test substrates such as foodstuffs, pharmaceuticals, and nutraceuticals, among others, on the gut microbiota in a closer approximation of *in vivo* physiological conditions.

## Limitations

Although we have gained some critical insights in this study in relation to optimised faecal fermentation media to use for *ex vivo* distal colon model experiments using the micro-Matrix bioreactor system, optimal duration of such experiments and whether pooled faecal samples or individual donor samples are more suitable, the present study does have some limitations. More specifically, since this study involved the use of four FFM compositions, several other media formulations reported in the literature have yet to be tested. Indeed, we endeavour to evaluate the performance of several other media compositions for suitability in the micro-Matrix system as an *ex vivo* distal colon model in the near future. Some of these media are designed to employ specific combinations of vitamin and mineral mixes that are often used for the growth of obligately anaerobic gut bacteria. It may very well be the case that other media compositions are even more suitable than the ones we have tested here with respect to mitigating microbial blooms and preserving microbial diversity in micro-Matrix experiments. Additionally, some key microbes of interest were only classified until the genus level, which we found to be the most useful taxonomic level for interpretation of our results in this study. However, deeper/metagenomic sequencing of samples may provide us species-level insights that may help us further improve these optimisations. Furthermore, we have only delved into a few specific genera of interest in this study; this may be expanded to a larger array of gut microbes of interest in the next iteration of method optimisation such as *Akkermansia, Eubacterium, Christensenella* and *Coprococcus*, among others. While MCF was definitely the best choice in maintaining the baseline populations of the gut microbes such as *Faecalibacterium, Roseburia* and *Bacteroides* studied in this work, there was still a general decline in their population over time. Other FFM might be better suited in this regard and will need to be evaluated in future studies. Ultimately, the main objective of any such future studies would be to either identify the most suitable media formulation and/or create a novel optimised media formulation by selecting appropriate ingredients from several types of existing media.

With regards to the generalizability of our main findings reported herein, further studies are warranted to ascertain whether the superior performance of MCF and MCD media found in our pilot study translates well to other populations and settings. Examples of such future studies may include the use of frozen slurries from specific cohorts of donors, e.g., infant faecal samples from cohorts less than 6 months old whereby the vast majority of the faecal microbiota may be composed of *Bifidobacterium* or faecal samples derived from specific disease cohorts whereby faecal microbiota profiles may show specific signatures of dysbiosis. Testing the suitability of MCF and MCD (as well as other media compositions described in the literature) in specific cohorts may reveal interesting insights into whether each of these compositions is more suitable for a certain cohort. Nonetheless, we hypothesise that the findings reported in this pilot study will translate reasonably well to studies involving the use of faecal slurries from a typical, non-patient, healthy, adult cohorts (as was the case in this study).

Beyond specific cohorts, it would also be interesting to ascertain whether our findings here translate well to other batch model systems such as MiniBio [21] and those described in other studies [22,23], as well as more complex semi-continuous and continuous model systems such as the SHIME [24] and TIM-2 models [25]. It must be noted however that typically in semi-continuous and continuous model systems, there is a dynamic influx and efflux of substrates/media and waste components, respectively, as well as the incorporation of an adaptation/stabilisation period for the faecal microbiota. Thus, the superior performance of certain media formulations described in this pilot study may or may not translate well to such systems. Nonetheless, it is hoped that the outcomes of our study will encourage other researchers using a variety of different model systems to evaluate the suitability of the different media formulations for their own specific objectives and research projects.

## Ethics statements

Informed consent was obtained from subjects who donated faecal samples for this study. This work was approved by the Clinical Research Ethics Committee, Cork Teaching Hospitals, University College Cork (APC163). In the process of seeking informed consent from potential volunteers, the privacy and confidentiality of the volunteers was maintained by contacting them individually in person or via email privately. Only the lead researcher (AM) had access to the signed informed consent forms from the volunteers. On the day of collection of the faecal samples from the donors, all faecal samples were pseudonymised by giving the samples arbitrary labels such as Donor 1, Donor 2 and such. Essentially, informed consent was obtained from 10 volunteers to prepare the pooled and individual donor faecal slurries, and 8 out of these 10 volunteers were able to donate samples on the day of generating the pooled and individual donor sample faecal slurries. The samples were deposited in a designated collection spot in the department and the samples were given arbitrary labels mentioned above. This was a double-blinded study whereby the investigators and the donors were unaware of which faecal sample came from which donor and this privacy and confidentiality was maintained throughout the course of this study. The study did not involve any animals.

## Declaration of competing interest

The authors declare the following financial interests/personal relationships which may be considered as potential competing interests: A.M., N.F.L., E.S., S.T. and H.M declare no competing interests. Research in P.D.C.’s laboratory has been funded by Friesland Campina, 10.13039/501100014710PrecisionBiotics Group, 10.13039/100004396PepsiCo and 10.13039/100007773Danone. P.D.C. has received support from PepsiCo, Yakult and H&H to attend/present at scientific meetings/conferences and is the CTO and a co-founder of SeqBiome Ltd.

## Data Availability

Data will be made available on request.

## Appendix Supplementary materials

Image, application 1

Image, application 2

Image, application 3
