# Supplementary material for: OPTIMATRIX v2.0: Optimised protocol to mitigate microbial blooms in the micro-Matrix bioreactor platform used as an ex vivo human distal colon model
Source: MethodsX. 2025 Mar 22;14:103275. doi: 10.1016/j.mex.2025.103275 (PMC11986543; doi:10.1016/j.mex.2025.103275)
Supplement: Supplementary file 1 [file mmc1.docx]

**Supplementary Information**

**OPTIMATRIX v2.0: optimised protocol to mitigate microbial blooms in the micro-Matrix bioreactor platform used as an *ex vivo* human distal colon model.**

Arghya Mukherjee^1,2^, Nicola Ferremi Leali^4^, Elisa Salvetti^5^, Sandra Torriani^4^, Paul D. Cotter**^1,2,3^, Harsh Mathur*^1,2^.

^1^ Teagasc Food Research Centre, Moorepark, Fermoy, Cork, Ireland.

^2^ APC Microbiome Ireland, Cork, Ireland.

^3^ VistaMilk, Fermoy, Co. Cork, Ireland.

^4^ Department of Biotechnology, University of Verona, Italy.

^5^ VUCC-DBT (Verona University Culture Collection, Dept. of Biotechnology), University of Verona, Italy.

* All correspondence should be addressed to: [harsh.mathur@teagasc.ie](mailto:harsh.mathur@teagasc.ie) AND paul.cotter@teagasc.ie

Twitter handle: @HarshMa47650720 AND @pauldcotter


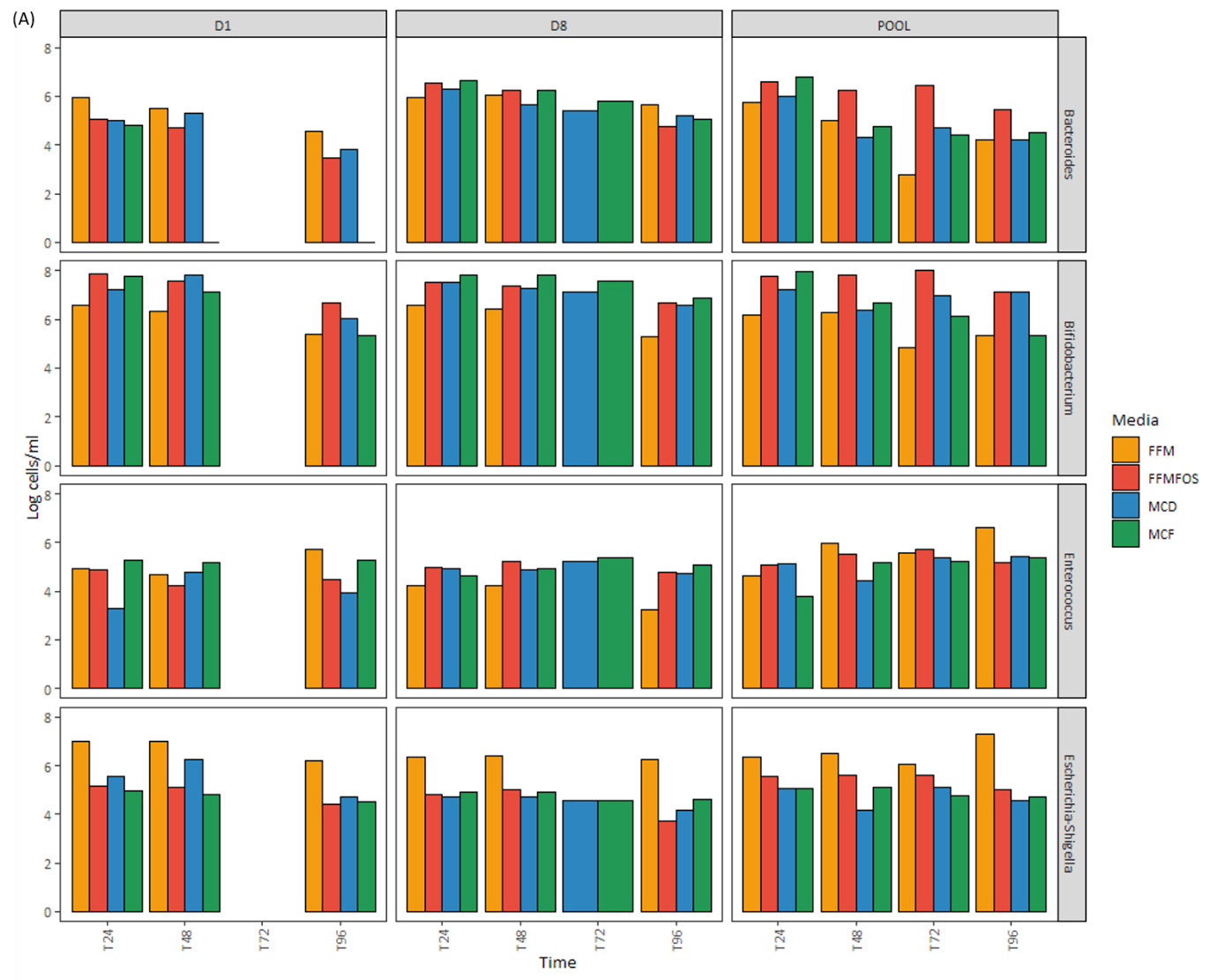


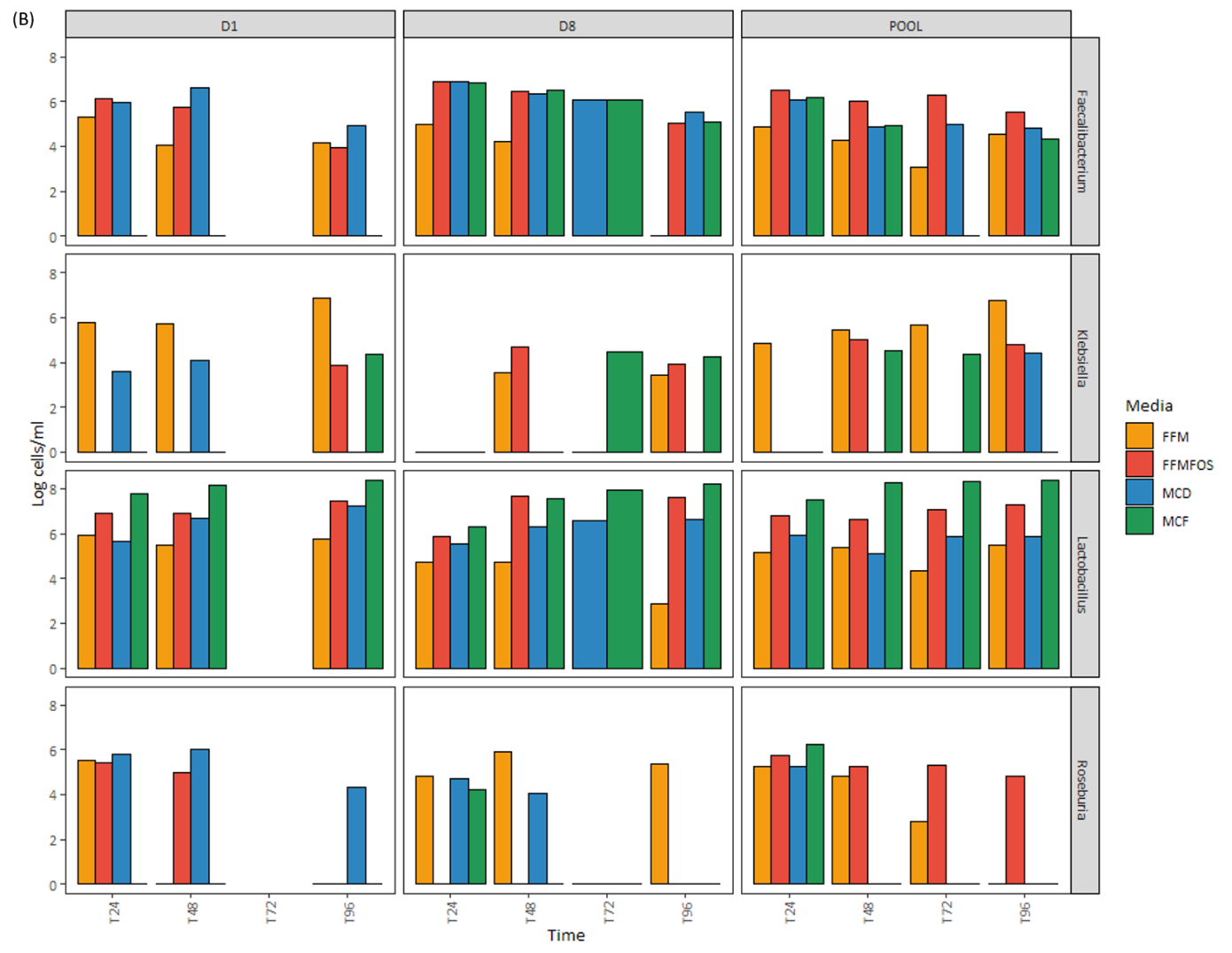


**Supplementary Figure S1. Absolute abundances for key genera of interest for faecal fermentation samples from Duran bottles used as an *ex vivo* distal colon model.** Absolute abundances are expressed as a logarithm of the cell counts obtained per ml (LCM) of the samples from flow cytometric estimations. T0 (start of experiment), T24 (24hrs into the experiment), T48 (48hrs into the experiment), T72 (72hrs into the experiment), T96 (96hrs into the experiment), T168 (end point of the experiment); BOT: Duran bottles; D1, Donor 1; D2; Donor 2; D5, Donor 5; D7, Donor 7; D8, Donor 8 and POOL, Pooled FSI from eight donors. FGM, Fooks & Gibson media; FGMFOS, Fooks & Gibson media supplemented with 2.5% w/v FOS; MCF, Modified media derived from MacFarlane et al. [5] MCD, Modified media derived from McDonald et al. [7]. For more information refer to Supplementary Table S4.

**
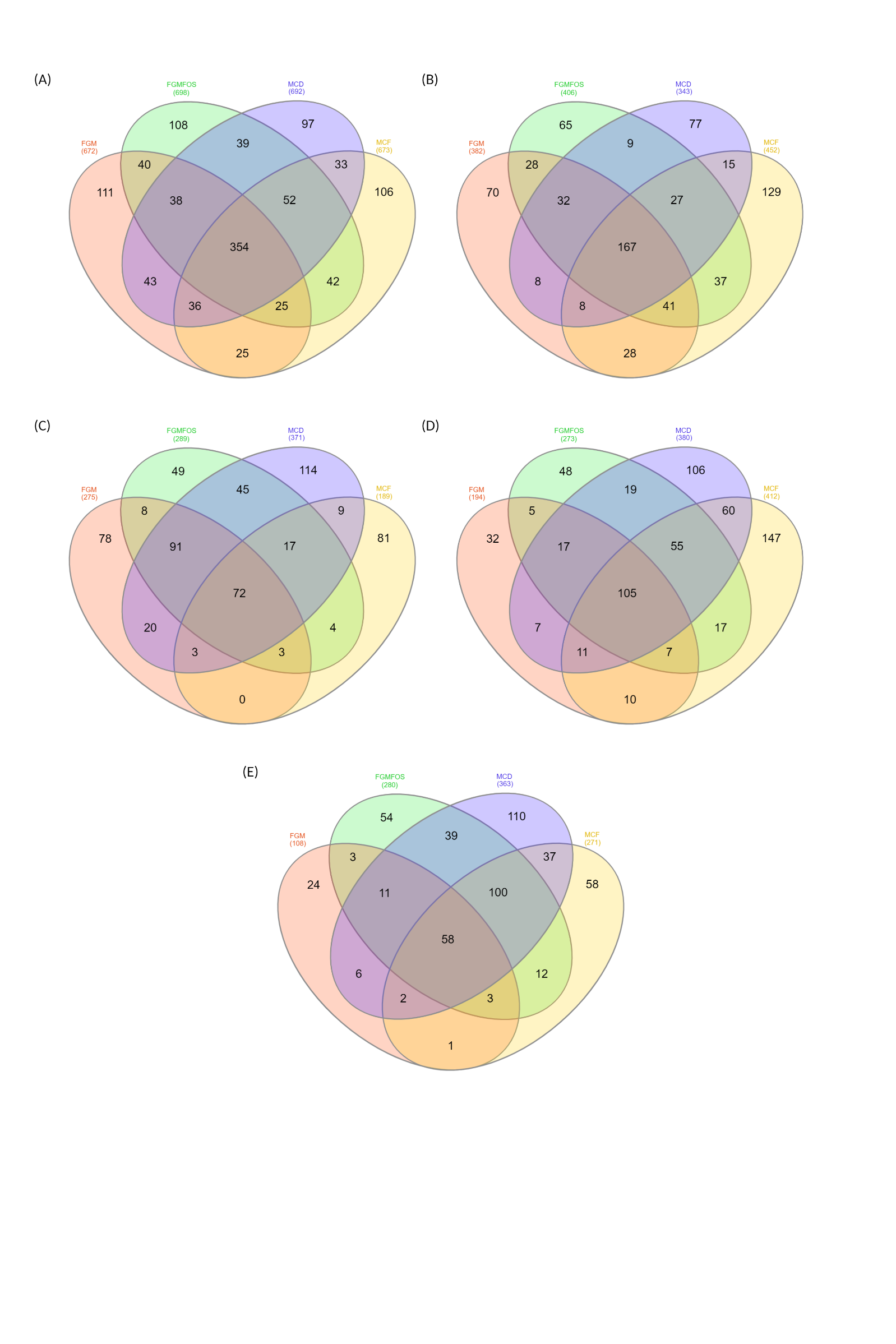
**

**Supplementary Figure S2. Shared and unique amplified sequencing variants (ASVs) between different media compositions in the micro-Matrix *ex vivo* distal colon model system.** The shared and unique ASVs (corresponding to ‘observed_features’) for each faecal fermentation medium in the micro-Matrix is shown using a Venn diagram (FGM: red; FGMFOS: green; MCD: blue; and, MCF: yellow). The datasets represented here are only for the pooled frozen standardised inoculum (FSI) at (A) T0, (B) T24, (C) T48, (D) T72, and, (E) T96.

**Materials and Methods**

*DNA extraction, quantification and normalisation*

DNA extractions were performed from each of the representative samples aliquoted from the micro-Matrix and corresponding experiments conducted in Duran bottles using the QiaAmp PowerFecal Pro kit (Qiagen, Hilden, Germany) following manufacturer’s instructions. Briefly, 1ml aliquots were taken from each of the micro-Matrix bioreactor wells at the relevant time points (T0, T24, T48, T72 and T96). One ml aliquots were also taken at the same time points from the corresponding Duran bottle experiments that were conducted in parallel. These aliquots were centrifuged at 16,000*g* for 10 minutes at room temperature to separate the faecal cell pellets and supernatants. The faecal cell pellets were then used for DNA extractions using the kit above following all the steps outlined by the manufacturer. DNA was eluted in volumes of 70µl at the final step. The extracted DNA was quantified using a Qubit 4.0 fluorometer (Invitrogen, Carlsbad, USA) and samples were diluted appropriately in 1 in 10 or 1 in 100 dilutions using 10mM Tris-HCl prior to normalisation to 5ng/µl as the starting concentration for 16S rRNA amplicon library preparations.

*16S rRNA amplicon library preparation and DNA sequencing*

Library preparations for 16S rRNA sequencing were conducted as described in the Illumina 16S Metagenomics Sequencing Library Preparation instructions and as outlined in Koc et al. [1]. DNA samples normalised to 5ng/µl were used as starting template concentrations to amplify the V3-V4 hypervariable regions of the 16S rRNA gene using the Forward primer: 5′ TCGTCGGCAGCGTCAGATGTGTATAAGAGACAGCCTACGGGNGGCWGCAG 3’ and Reverse primer: 5’ GTCTCGTGGGCTCGGAGATGTGTATAAGAGACAGGACTACHVGGGTATCTAATC 3’ as recommended by Illumina and as outlined in Koc et al. [1] . This is expected to produce an amplicon size of ~444 bp. Primers were purchased from Sigma Aldrich (Vale Road, Arklow, Ireland). A 2627 Thermo Cycler (Life Technologies, MA, USA) was used for PCR reactions and KAPA HiFi Hot Start Ready-mix (Kapa Biosystems, MA, USA) was used as the polymerase for these library preparations. The following programme was used for the amplicon PCR: initial denaturation at 95°C for 5 minutes followed by 30 cycles of 95°C for 30 seconds, 55°C for 30 seconds and 72°C for 30 seconds. After the 30 cycles, a final extension step of 72°C for 5 minutes was used. For indexing, identical PCR conditions were used with the only exception being that 8 cycles were used for the index PCR instead of the 30 cycles used in the amplicon PCR. The Nextera XT set of indices (Illumina Inc. San Diego, USA) were used for unique index combinations for each of the samples in the library. PCR amplicons were visualised in a 2% agarose gel (Sigma Aldrich, Vale Road, Arklow, Ireland) stained with Midori Green (Thermo Fisher, MA, USA). AMPure XP beads (Beckman Coulter, CA, USA) were used for the clean-up steps in the library preparation following instructions in the protocol outlined by Illumina. Finally, libraries were normalised to 4nM final concentrations prior to pooling and were sequenced in the Teagasc Next Generation DNA Sequencing Facility using an Illumina NextSeq 2000 P1 600 cycle (2 X 300bp) reagent kit (Illumina Inc. San Diego, USA).

*Bioinformatic analysis*

16S rRNA amplicon data for all samples were checked for quality using FastQC and MultiQC [2, 3]. 16S rRNA sequences were then processed using QIIME2 [4]. Briefly, demultiplexed 16S rRNA sequences were denoised using the QIIME2 implementation of DADA2 [5], with truncation and trimming of the sequences carried out using the following arguments (--p-trim-left-f 10 --p-trim-left-r 10 --p-trunc-len-f 290 --p-trunc-len-r 220). Amplicon sequencing variants (ASVs) thereby identified using DADA2 were then taxonomically classified using the SILVA SSU r138.2 release [6] in QIIME2 and annotations for chloroplasts and mitochondria removed. Compositional taxonomic data for samples were then extracted for each taxonomic level and graphed as relative abundances with ggplot2 in R [7]. 16S rRNA datasets were rarefied through subsampling (n=28,460), which in turn were used to obtain the ‘observed_features’ (number of amplicon sequencing variants) metric for each sample. This information was further used to chart the unique and shared amplicon sequencing variants using InteractiVenn [8]. Shared and core features in the dataset was identified using the ‘core_features’ command in QIIME2.

*Flow cytometry and absolute abundance quantifications*

Flow cytometry was conducted using a BD Accuri C6 flow cytometer (Beckton Dickinson, Belgium) described previously [9, 10] with a few minor modifications. Since the BD Accuri C6 flow cytometer at Teagasc, Moorepark, is primarily dedicated for class I bacterial samples, the samples from the micro-Matrix experiments and corresponding Duran bottle experiments were autoclaved prior to flow cytometry analysis in order to heat-kill the bacterial cells present in the faecal samples. Briefly, 500 µl aliquots taken from each bioreactor well and corresponding Duran bottle experiments and autoclaved at 121°C for 15 minutes. This rendered all the bacterial cells inactive, thereby enabling us to quantify the total number of bacterial cells present within the corresponding aliquots, as cell viability was not a requirement for the purposes of absolute abundance quantifications which was the main objective of flow cytometry analysis conducted here in this study. The BacLight Bacterial Viability kit (Life Technologies, Carlsbad, USA) with Syto 9 and Propidium Iodide was used for staining and subsequent determination of the total number of bacterial cells present.

In order to stain an appropriate number of bacterial cells for flow cytometry, 1 in 10 serial dilutions of the autoclaved aliquots were conducted using phosphate buffered saline (PBS) as a diluent. This was with a view to staining in the 100 to 1,000 cellular events per minute range in order to minimise the phenomenon of coincidence in the flow cytometer. A volume of 125 µl of the appropriate dilution was stained using the BacLight bacterial viability kit (Life Technologies, Carlsbad, USA) using a concentration of 6.68 µM Syto 9 and 40 µM PI at 37°C for 10 minutes in the dark with agitation in a shaking heating block at 400rpm. Since autoclaving rendered the bacterial cells inactive, PI was able to displace Syto 9 in the dual staining technique due to its higher binding affinity for nucleic acids. These stained samples were subjected to a medium flow rate of 35 µl per minute and a threshold of 10,000 on the Forward Scatter (FSC) settings was applied in order to minimise background due to potential cell debris. Acquisition and downstream analysis of Syto 9 (FL1) and PI fluorescence (FL3, > 670nm long pass) was conducted using the BD Accuri C6 software version 1.0.2 (Beckton Dickinson, Belgium). The microbial populations were identified on the basis of their scattering properties using forward scatter (FSC) versus side scatter (SSC) bi-plots and a quadrant style gating strategy was applied to discriminate the microbial population from cell debris and background particles. Absolute abundance was calculated by merging data relating to the total number of bacterial cells in each sample with the relative abundance data as described previously in Mathur et al. 2023.

**References**

1. F. Koç, I. Sugrue, K. Murphy, S. Renzetti, M. Noort, R.P. Ross, C. Stanton, The microbiome modulating potential of superheated steam (SHS) treatment of dietary fibres, Innovative Food Science & Emerging Technologies, 80 (103082) (2022), doi: 10.1016/j.ifset.2022.103082.
2. S. Andrews. FastQC: A Quality Control Tool for High Throughput Sequence Data (Online). 2010.
3. P. Ewels, M. Magnusson, S. Lundin, M. Käller, MultiQC: summarize analysis results for multiple tools and samples in a single report, Bioinformatics, 32(19), (2016), 3047-8.doi:10.1093/bioinformatics/btw354.
4. E. Bolyen, J. R. Rideout, M. R. Dillon, N. A. Bokulich, C. C. Abnet, G. A. Al-Ghalith, H. Alexander, E. J. Alm, M. Arumugam, F. Asnicar, Y. Bai, J. E. Bisanz, K. Bittinger et al. Reproducible, interactive, scalable and extensible microbiome data science using QIIME 2, Nat. Biotechnol. 37(8), (2019), 852-857, doi: 10.1038/s41587-019-0209-9.
5. B. J. Callahan, P. J. McMurdie, M. J. Rosen, A. W. Han, A. J. A. Johnson, S. P. Holmes, DADA2: High-resolution sample inference from Illumina amplicon data, Nature Methods, (13), (2016), 581–583, doi: 10.1038/nmeth.3869.
6. E. Pruesse, C. Quast, K. Knittel, B. M. Fuchs, W. Ludwig, J. Peplies, F. Glöckner. SILVA: a comprehensive online resource for quality checked and aligned ribosomal RNA sequence data compatible with ARB, Nucleic Acids Research, 35(21), (2007), 7188–7196, doi: 10.1093/nar/gkm864.
7. H. Wickham. ggplot2. Elegant Graphics for Data Analysis. 2016.
8. H. Heberle, G. V. Meirelles, F. R. da Silva, G. P. Telles, R. Minghim. InteractiVenn: a web-based tool for the analysis of sets through Venn diagrams, BMC Bioinformatics, 16(1), 169, (2015), doi: 10.1186/s12859-015-0611-3.
9. H. Mathur, V. Fallico, P.M. O'Connor, M.C. Rea, P.D. Cotter, C. Hill, R.P. Ross, Insights into the Mode of Action of the Sactibiotic Thuricin CD, Front. Microbiol. 8:696, (2017), doi: 10.3389/fmicb.2017.00696.
10. D. Field, T. Blake, H. Mathur, P.M. O' Connor, P.D. Cotter, R.P. Ross, C. Hill, Bioengineering nisin to overcome the nisin resistance protein, Mol. Microbiol. 111(3), (2019), 717-731, doi: 10.1111/mmi.14183.
