# Supplementary figures and images for: OPTIMATRIX v2.0: Optimised protocol to mitigate microbial blooms in the micro-Matrix bioreactor platform used as an ex vivo human distal colon model
Source: MethodsX. 2025 Mar 22;14:103275. doi: 10.1016/j.mex.2025.103275 (PMC11986543; doi:10.1016/j.mex.2025.103275)

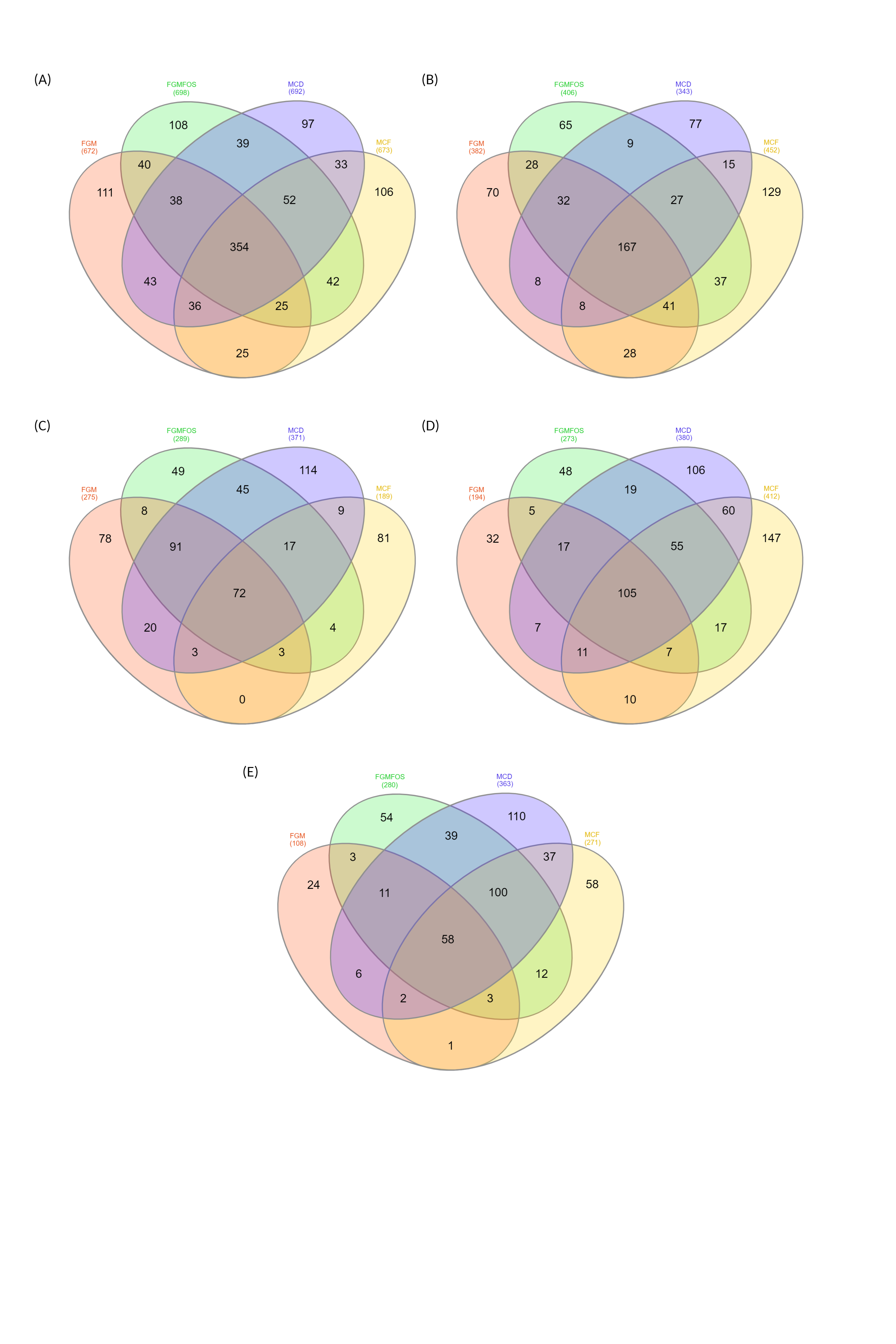

Supplement: Supplementary file 2 [file mmc2.zip › 110325 Mukherjee et al revised Figure S2.png]
